# Genetic and phenotypic analysis of the causal relationship between aging and COVID-19

**DOI:** 10.1038/s43856-021-00033-z

**Published:** 2021-10-05

**Authors:** Kejun Ying, Ranran Zhai, Timothy V. Pyrkov, Anastasia V. Shindyapina, Marco Mariotti, Peter O. Fedichev, Xia Shen, Vadim N. Gladyshev

**Affiliations:** 1 Biostatistics Group, State Key Laboratory of Biocontrol, School of Life Sciences, Sun Yat-sen University, Guangzhou, China; 2 Division of Genetics, Department of Medicine, Brigham and Women’s Hospital and Harvard Medical School, Boston, MA USA; 3 T. H. Chan School of Public Health, Harvard University, Boston, MA USA; 4 Gero LLC PTE, Singapore City, Singapore; 5 Department of Genetics, Microbiology and Statistics, Universitat de Barcelona, Barcelona, Catalonia Spain; 6 Moscow Institute of Physics and Technology, Dolgoprudny, Moscow Region Russia; 7 Greater Bay Area Institute of Precision Medicine (Guangzhou), Fudan University, Guangzhou, China

**Keywords:** Viral infection, Disease genetics, Ageing

## Abstract

**Background:**

Epidemiological studies revealed that the elderly and those with comorbidities are most affected by COVID-19, but it is important to investigate shared genetic mechanisms between COVID-19 risk and aging.

**Methods:**

We conducted a multi-instrument Mendelian Randomization analysis of multiple lifespan-related traits and COVID-19. Aging clock models were applied to the subjects with different COVID-19 conditions in the UK-Biobank cohort. We performed a bivariate genomic scan for age-related COVID-19 and Mendelian Randomization analysis of 389 immune cell traits to investigate their effect on lifespan and COVID-19 risk.

**Results:**

We show that the genetic variation that supports longer life is significantly associated with the lower risk of COVID-19 infection and hospitalization. The odds ratio is 0.31 (*P* = 9.7 × 10^−6^) and 0.46 (*P* = 3.3 × 10^−4^), respectively, per additional 10 years of life. We detect an association between biological age acceleration and future incidence and severity of COVID-19 infection. Genetic profiling of age-related COVID-19 infection indicates key contributions of Notch signaling and immune system development. We reveal a negative correlation between the effects of immune cell traits on lifespan and COVID-19 risk. We find that lower B-cell CD19 levels are indicative of an increased risk of COVID-19 and decreased life expectancy, which is further validated by COVID-19 clinical data.

**Conclusions:**

Our analysis suggests that the factors that accelerate aging lead to an increased COVID-19 risk and point to the importance of Notch signaling and B cells in both. Interventions that target these factors to reduce biological age may reduce the risk of COVID-19.

## Plain Language Summary

Older adults and those with comorbidities are more likely to develop severe COVID-19 if infected with SARS-CoV-2. In this study, we investigate the genetic factors underlying the link between aging and COVID-19. Using data on the genetic variation between individuals and statistical methods to allow us to determine causality, we find that genetic variation associated with longer lifespan is associated with reduced risk of COVID-19 infection and hospitalization. We also find that acceleration of biological age (i.e., the age of your body based on physiological measurements rather than time) is associated with future incidence and severity of COVID-19, and identify some of the key cells and molecules involved in aging-related COVID-19 risk. Our study helps to characterize the relationship between aging and COVID-19, which may help to identify strategies to protect or treat older adults.

## Abstract (web summary)

Ying et al. conduct a multi-instrument Mendelian randomization study looking at the link between aging and COVID-19 risk. They observe an association between genetic variation implicated in longevity and decreased risk of COVID-19 infection and hospitalization, with Notch signaling and immune system development loci found to be important in aging-related COVID-19 risk.

## Introduction

The coronavirus disease 2019 (COVID-19), caused by severe acute respiratory coronavirus 2 (SARS-CoV-2), first emerged in late 2019 and has led to an unprecedented global health crisis^1^. Notably, the aging population is particularly at risk of COVID-19^2^, e.g., in Italy, 88% of the individuals who tested positive for COVID-19 were 40 years or older^3^. A recent report based on epidemiological data from multiple countries showed that 69% of infections in people over 70 are symptomatic, whereas this number drops to 21% for 10−19-year-olds^4^. Unsurprisingly, elderly people are also more likely to die from COVID-19, and the case fatality rate for COVID-19 grows exponentially with age^3^. As observational evidence implies a strong link between COVID-19 and age, COVID-19 can be considered a disease of aging^3^, and multiple clinical trials using potential lifespan-extending drugs (e.g., metformin, rapamycin, and senolytics) to protect the elderly from COVID-19 have been proposed^5–7^. Although observational data on metformin seems promising^8,9^, it is unclear if other lifespan-extending drugs should be prioritized in clinical trials since the evidence of any causal link between lifespan and COVID-19 susceptibility is still missing.

Mendelian Randomization (MR) is a genetic instrumental variable approach that assesses the causal effect of exposure of interest on an outcome by ascertaining genetic variants, e.g., single nucleotide polymorphisms (SNPs), strongly associated with the exposure phenotype. Since the alleles of the genetic variants are naturally randomly allocated at conception, when the genetic effects on the outcome are only mediated through the exposure, the causal effect inferred by MR is, in analogy to randomized clinical trials (RCTs), free of any environmental confounders and reverse causation. Although RCTs are considered a gold standard for establishing causal relationships, MR can provide valuable insights into causality when it is not feasible to perform an RCT or before an RCT is performed^10^.

In this study, we perform a multi-SNP MR analysis to elucidate whether and how the rate of aging is associated with COVID-19. We consider four lifespan-related traits (parental lifespan, healthspan, longevity, and healthy aging (the combination of these three traits)) as exposures and evaluate their causal effects on COVID-19 infection and related phenotypes. To support the argument, we also estimate the biological age acceleration in COVID-19 patients from the UK Biobank (UKBB) cohort and observe a significant association between the phenotypic indicators of aging progress (aging clocks) and the risk and case fatality rate of COVID-19. To provide functional insight into how aging contributes to a higher risk of COVID-19, we conduct a bivariate genomic scan to highlight the loci contributing to both aging and COVID-19 risk, identifying the Notch signaling pathway and immune system development. Finally, we perform MR using 389 immune cell traits as exposure and observe a significant negative correlation between their effect on lifespan and COVID-19 risk, especially for B cell-related traits. More specifically, we discover that lower CD19 levels in B cells may increase the risk of COVID-19 and decrease lifespan, which is further validated by clinical data from COVID-19 subjects.

## Methods

### GWAS data for lifespan-related traits and diseases

We studied four lifespan-related traits (lifespan, longevity, healthspan, and a combined trait) with publicly available GWAS summary statistics. The parental lifespan GWAS included unrelated, European-ancestry subjects (a total of 512,047 mothers’ and 500,193 fathers’ lifespan), 60% of which were complete. The statistics for every cohort were calculated by fitting Cox survival models to mother’s and father’s survival, respectively, taking account of 10 principal components, study-specific covariates, and sex. In the GWAS setting, parental lifespan is the same phenotype as the general lifespan of individuals (but with a weaker power) due to the fact that the genetic effect on a parental phenotype is simply half of the individual’s phenotype itself. Thanks to the large sample size of UK Biobank, such a GWAS is powerful enough to uncover the genetic architecture^11^.

The longevity GWAS included unrelated, European-ancestry subjects with a lifespan above the 90th survival percentile (*N* = 11,262) or whose age at the last follow-up visit (or age at death) was before the 60th percentile age (*N* = 25,483). The statistics for each cohort were calculated using logistic regression and then combined using a fixed-effect meta-analysis^12^. The healthspan GWAS contained 300,477 unrelated, British-ancestry individuals from UKBB. The statistics were calculated by fitting Cox−Gompertz survival models. The healthspan was defined as the age of the first incidence of dementia, congestive heart failure, diabetes, chronic obstructive pulmonary disease, stroke, cancer, myocardial infarction, or demise^13^.

The summary association statistics of healthy aging was from the meta-analysis of healthspan, lifespan, and longevity summary statistics using MANOVA^14^, while accounting for correlations between studies due to sample overlap and correlation amongst the traits. Summary association statistics were calculated for 7,320,282 SNPs shared between the studies. These statistics represented the significance of each SNP affecting one or more of the traits, giving a *P*-value against the null hypothesis that effect sizes are zero in all studies^14,15^.

We investigated four additional traits genetically correlated with lifespan using published case-control studies: Alzheimer’s disease^16^, coronary artery disease (CAD)^17^, type 2 diabetes^18^, and smoking^19^ (Table S1).

We also included GWAS for age acceleration measured by four epigenetic clocks, including Hannum age, Horvath age, PhenoAge, and GrimAge^20^. The epigenetic age was estimated for 34,449 healthy individuals of European ancestry. In addition to epigenetic age, we include two physical function-related traits, the pace of walk and the sedentary lifestyle, as they are correlated with the rate of aging and therefore can serve as the surrogates to the biological age^21,22^.

GWAS data for 22 common diseases were from a community-based study, Genetic Epidemiology Research on Adult Health and Aging (GERA)^23^. There were 60,586 individuals of European ancestry in the GERA data. The summary statistics of these diseases were adjusted with age, gender, and the first 20 PCs.

We used 1000 Genomes Phase 3 reference (released in 2014 October) to map variants in the GWAS results to rsIDs by chromosome, position, and alleles. Only the autosomal SNPs available in the 1000 Genomes reference panel were used, and the 1000 Genomes European ancestry reference was used to estimate the linkage disequilibrium (LD) among these SNPs. Duplicated rsIDs in the data were removed prior to the analysis.

### COVID-19-related traits

To extensively evaluate the genetic effects on COVID-19 risk, we used GWAS summary statistics data from 8 COVID-19-related traits (Table S1). The GWAS results for SARS-COV-2 infection are from the National Institute of Health, Genome-Wide Repository of Associations Between SNPs and Phenotypes (NIH-GRASP), which includes 1,503 positive cases and 11,409 negative or 457,747 UK Biobank controls with European ancestry; the GWAS summary statistics for the critical illness was from the GenOMICC (Genetics Of Mortality In Critical Care) study in 2,244 critically ill Covid-19 patients from 208 UK intensive care units^24^. The rest of the five traits are from the COVID-19 Host Genetics Initiative (HGI) release 5 (Jan 2021), with the sample size varies from 1,332 to 1,079,768^25^. Those traits including COVID-19 hospitalization (versus non-hospitalized COVID-19 or population control), susceptibility (affected versus unaffected population), very severe respiratory confirmed COVID-19 (versus the general population), and COVID-19 infection (versus population).

### Expression quantitative trait loci (eQTLs) and age-related gene expression in blood

Blood eQTL data were obtained from the eQTLGen Consortium (31,684 whole blood samples)^26^. Only the significant near-independent eQTLs (FDR-q < 0.05, *r*^2^ < 0.05) were used in the MR analysis.

The age-related transcriptomic change in whole blood was obtained from a large-scale meta-analysis^27^, including six European-ancestry studies (*n* = 7,074 samples), and detected roughly half of the genes in the human genome (*n* = 11,908). The direction and *P*-value of age-related differential expression were directly obtained from the published dataset.

### Immune cell traits

The GWAS summary statistics of immune cell-type-specific surface marker levels are obtained from the largest immune cell GWAS study^28^. 389 median fluorescence intensities (MFIs) of surface antigens were profiled by flow cytometry and assessed in a general population cohort of 3,757 Sardinians.

### Mendelian randomization analysis

MR is a method that uses genetic variants as instrumental variables to determine whether an observational association between a risk factor and an outcome is consistent with a potential causal effect^29^. The multi-SNP MR analysis was implemented using GSMR (Generalized Summary-data-based MR) in GCTA^30^.

As instruments for each exposure (four lifespan-related traits, four risk factors, and four epigenetic age acceleration traits), we selected near-independent SNPs (*r*^2^ < 0.1) with genome-wide significant (*P* < 5 × 10^−8^) association with the exposure. For the expression of *NOTCH1-4* in whole blood and other tissues, we selected significant near-independent eQTLs (FDR-q < 0.05, *r*^2^ < 0.05); For 22 diseases from GERA community-based study, we selected SNPs with suggestive genome-wide significance (*P* < 1 × 10^−6^) as instruments and performed a separate analysis due to the limited case number in the community-based study. A full list of genetic instruments is provided (Supplementary Data 1).

GSMR includes a HEIDI-outlier filter to remove potential pleiotropic SNPs that affect the exposures and the outcomes via different pathways. We set its *p*-value threshold to 0.01 and tested the remaining SNPs for association with the COVID-19-related traits. The required minimum number of instrumental SNPs for each exposure in the analysis was lowered to 1.

### Conditional analysis

To test whether the effect of lifespan-related traits on COVID-19 risk depends on certain age-related diseases and *vice versa*, we performed a conditional analysis using a two-step approach, as described by Zhu et al.^30^. In the first step, we performed a conditional GWAS analysis to adjust the exposure of interest with other risk factors using mtCOJO (multi-trait-based conditional and joint analysis). In the second step, we estimate the effect of adjusted exposure on the outcome using GSMR as previously described. We, therefore, can estimate the effect size of lifespan-related traits on COVID-19, accounting for other age-related risk factors by a GSMR analysis using SNP effects conditioning on covariate traits. Notably, as the exposures are very highly correlated, the multivariate MR will have lower power. To estimate the causal effects of conditional traits, we had to lower the *P*-value threshold for genetic instruments to 5e−6 to obtain a sufficient number of SNPs for MR analysis. To make the univariate and conditional analysis results comparable, we also performed a univariate analysis using the same *P*-value threshold.

### Sensitivity analysis

We used GSMR for the main analyses because it gains power by taking account of sampling variation of the effect size of SNPs on exposure and outcome, compared with the MR-Egger and inverse variance weighted (IVW) methods^30^. GSMR also accounts for the remaining LD among instruments after clumping analyses. To compare the results from other MR methods based on various assumptions, we performed a sensitivity analysis using the Maximum likelihood method^31^, MR-Egger method^32^, and simple median method^33^.

The Maximum likelihood method estimates the causal effect by maximization of the likelihood based on the effect of SNPs on exposure and outcome. It gives robust estimates even in the presence of small measurement error for the effect of SNPs on exposure^31^; the MR-Egger method is the modification of the IVW method, which allows a non-zero intercept. This way, it allows unbalanced pleiotropic effects across all of the instruments while still returns unbiased causal effect estimates. This method assumes no correlations between horizontal pleiotropic effects and SNP-exposure effects (the InSIDE assumption)^32^. The MR-Egger regression also provides an intercept test to detect the directional pleiotropy in the instruments (i.e., the pleiotropic effect is evident if the intercept term significantly deviates from 0). Lastly, the simple median method takes the median effect of all instrumental SNPs. It only requires half of the SNPs to be valid to return accurate causal effect estimates.

We selected independent instrumental SNPs (*r*^2^ < 0.01) for each exposure with the same genome-wide significance threshold as in GSMR analysis (*P* < 5 × 10^−8^ for lifespan-related traits and FDR < 0.05 for eQTLs); the analysis was then performed using the “TwoSampleMR” R package (https://mrcieu.github.io/TwoSampleMR)^34^.

### Bivariate genomic scan and functional annotation

To identify genetic variants associated with aging-related COVID-19 risk, we meta-analyzed UKBB COVID-19 infection (with population control) and healthy aging (with the sign of effect size reversed) summary statistics while accounting for correlations between studies due to sample overlap and correlation between the traits, as implemented in MultiABEL v1.1-610^14,35^. Summary association statistics were calculated for the 7,318,649 SNPs shared between studies. These statistics represent the significance and consistency of each SNP affecting one or both of the traits (e.g., the SNPs that significantly contribute to aging and COVID-19 risk in the same direction will have a smaller *P*-value). Therefore, we refer to this bivariate genomic scan result as the aging-related COVID-19 throughout this study.

We then used the summary statistics of aging-related COVID-19 and performed functional annotation for all SNPs in genomic areas identified by lead SNPs (*P* < 1 × 10^−6^, 250 Kb apart) using FUMA (Functional Mapping and Annotation)^36^. The annotated genes were used for functional enrichment analyses using the default setting of the FUMA platform.

### Genetic correlation analysis

We estimated genetic correlations between lifespan-related traits, risk factors, epigenetic age acceleration, and COVID-19 using LD score regression (LDSC) and high-definition likelihood (HDL) methods^37,38^. SNPs that are imperfectly imputed (INFO < 0.9) or with low frequency (MAF < 0.05) were removed to reduce statistical noise. LDSC was performed using LDSC software v1.0.1 (https://github.com/bulik/ldsc); the HDL was performed using R package “HDL” v1.3.8 (https://github.com/zhenin/HDL).

### Biological age estimation for UKBB cohorts

The collection of the UK Biobank (UKBB) data was approved by the UKBB’s Research Ethics Committee. Access to the UK Biobank data was granted for this work under UK Biobank application number 21988. All-cause mortality increases exponentially with age. Hence, log-linear risk predictors from proportional hazards models can provide natural composite organism state representations characterizing the progression of aging based on biological and physiological measurements. We used two such biological age measures: Phenotypic Age based on blood biochemistry^39^ and Dynamic Organism State Indicator (DOSI) based on widely available Complete Blood Counts (CBC)^40^. The latter is a proxy for the frailty index and is derived from the blood markers only, whereas the Phenotypic Age additionally employs the explicit age. We also used physical activity (number of steps per day averaged over the week), which is associated with all-cause mortality and hence can also be viewed as a measure of biological aging^41^.

We investigated an association between the incidence of COVID-19 and biological age acceleration (which is the difference between the biological age of a person and the average biological age in the cohort of individuals of the same age and sex) using logistic regression. Chronological age and biological sex were used as additional covariates in the analysis.

Following UKBB recommendations, we used the “result” label from the table “COVID-19 test results table” as the proxy of disease severity. This implies that primarily those individuals that showed characteristic COVID-19 symptoms were selected for testing. We investigated biological age acceleration associations with the incidence of COVID-19 and its associated fatality using all available cases (All) and separately cohorts of individuals who have (Frail) or do not have (Not Frail) major chronic diseases (from the list including all kinds of cancer, angina pectoris, coronary heart disease, heart attack, heart failure, hypertension, stroke, diabetes, arthritis, bronchitis, and emphysema) at the time of infection.

### Reporting summary

Further information on research design is available in the Nature Research Reporting Summary linked to this article.

## Results

### Genetic and MR analysis of lifespan-related traits and COVID-19 risk

We applied MR using GSMR to test for potential causal associations between four lifespan-related traits and COVID-19, including lifespan, longevity (i.e., surviving to the 90th percentile), healthspan (time to a first major age-related disease), and healthy aging (multivariate meta-analysis of all three traits combined) (Table S1). We employed summary-level GWAS data^11–13,15^ and selected near-independent SNPs at a genome-wide significance level as genetic instruments for each trait. The HEIDI-outlier filter was used to detect and eliminate genetic instruments with pleiotropic effects on both exposure and outcome, as described by Zhu et al^30^. For the outcomes, we used eight different sets of GWAS summary statistic data for COVID-19-related traits from case-control studies (Table S1).

**Fig. 1:**
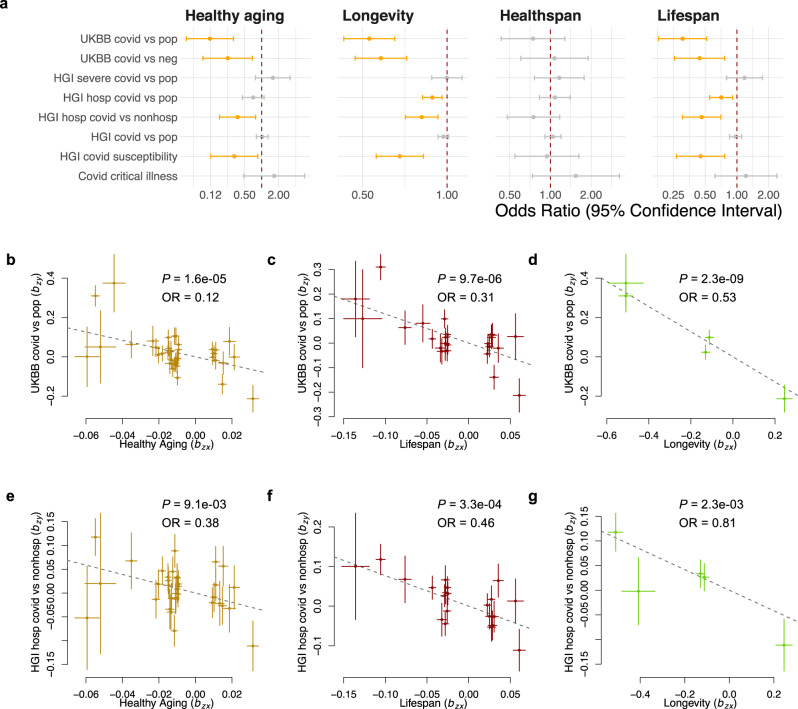
Mendelian randomization analysis reveals an association of lifespan-related traits with the risk of COVID-19. **a** Forest plot shows Mendelian randomization estimates for the causal effect of lifespan-related traits on the risk of COVID-19. Error bars show 95% confidential interval. Significant effects with FDR < 0.05 are in orange. Nominally significant effects (*P* < 0.05) are in black. Plots of effect sizes of all genetic instruments from GWAS for healthy aging (**b**), lifespan (**c**), and longevity (**d**) (*x*-axis) versus those for UKBB COVID-19 (*y*-axis); and the same set of exposure traits (**e**−**g**) (*x*-axis) versus HGI COVID-19 hospitalization (*y*-axis). Error bars represent standard errors. UKBB UK Biobank, HGI Host Genetics Initiative, pop population control, OR Odds Ratio.

Strikingly, our MR analysis showed that genetic variants associated with longer lifespan, longevity, and healthy aging are both protective against COVID-19 infection and lowered the chance of being hospitalized after getting COVID-19 (Fig. 1a−g and Table 1). For lifespan, the estimated odds ratio of being infected by SARS-CoV-2 was 0.31 (95% CI: 0.18−0.52; *P* = 9.7 × 10^−6^), indicating that the risk of infection is decreased by 69% with approximately every additional ten years of life; similarly, the risk of getting hospitalized after being infected with SARS-CoV-2, which is usually due to the development of severe symptoms, was also decreased by 54% (OR 95% CI: 0.18−0.52; *P* = 3.3 × 10^−4^) with every additional ten years of predicted lifespan. For the longevity trait, the risk of COVID-19 infection and hospitalization was decreased by 47% (OR 95% CI: 0.43−0.65; *P* = 2.3 × 10^−9^) and 19% (OR 95% CI: 0.71−0.93; *P* = 2.3 × 10^−3^), respectively, with each unit higher log odds of surviving to the 90th percentile in the population. None of the lifespan-related traits showed a significant protective effect on COVID-19 with a severe respiratory disorder or critical illness, possibly due to the small case number of severe COVID-19 (Table S1).

**Table 1:** Mendelian randomization estimates for the association between lifespan-related traits and risk of COVID-19.

Exposure	Outcome	OR	95% CI	*P*
Healthy aging	HGI covid susceptibility	0.33	0.13−0.85	2.2e−02
Healthy aging	HGI hosp covid vs. nonhosp	0.38	0.18−0.78	9.1e−03
Healthy aging	UKBB covid vs. neg	0.25	0.09−0.69	7.0e−03
Healthy aging	UKBB covid vs. pop	0.12	0.05−0.32	1.6e−05
Longevity	HGI covid susceptibility	0.68	0.56−0.82	8.5e−05
Longevity	HGI hosp covid vs. nonhosp	0.81	0.71−0.93	2.3e−03
Longevity	HGI hosp covid vs. pop	0.89	0.82−0.96	2.9e−03
Longevity	UKBB covid vs. neg	0.58	0.47−0.72	5.1e−07
Longevity	UKBB covid vs. pop	0.53	0.43−0.65	2.3e−09
Lifespan	HGI covid susceptibility	0.45	0.27−0.77	3.2e−03
Lifespan	HGI hosp covid vs. nonhosp	0.46	0.3−0.71	3.3e−04
Lifespan	HGI hosp covid vs. pop	0.71	0.55−0.91	6.8e−03
Lifespan	UKBB covid vs. neg	0.44	0.26−0.77	3.6e−03
Lifespan	UKBB covid vs. pop	0.31	0.18−0.52	9.7e−06

Only the associations that reached nominal significance (*P* < 0.05) are shown. hosp: hospitalized COVID-19 patient; nonhosp: non-hospitalized COVID-19 patient; pop: population control; neg: COVID-19 negative control.

The GSMR was used as the main analysis because it gains power by taking account of sampling variation of the effect size of SNPs on exposure and outcome, compared with the MR-Egger method and IVW method^30^. To further investigate the robustness of our findings, we performed a sensitivity analysis using multiple MR methods, which can provide a reliable estimate of the causal effect even with invalid SNPs (i.e., horizontal pleiotropy or measurement error of the SNP-exposure effect), at the cost of lower power (see “Methods”). The protective effect of genetically proxied lifespan on the risk of COVID-19 infection was consistently estimated using the Maximum likelihood method, the MR-Egger method, and the simple median method with largely overlapped 95% CI, and only for the simple median method the 95% CI included the null (Fig. S1). The findings for longevity are also consistent across all methods, with only the MR-Egger method giving a 95% CI crossing the null. Likewise, the MR sensitivity analysis for COVID-19 hospitalization produced similar estimates to the main analysis, only with a wider 95% CI that crossed the null (Fig. S1).

To further examine the pleiotropic effect across the instruments used in the MR analysis, we tested for the directional pleiotropy using the intercept term of MR-Egger regression. The MR-Egger intercept terms do not differ from zero (*P* > 0.1) for most of the significant exposure-outcome pairs identified in the main analysis, suggesting there is no imbalanced pleiotropic effect (Table S2). The only exception is for the association between lifespan and COVID-19 infection compared with the population, for which the Egger intercept is 0.07 (*P* = 0.04). However, this does not affect the validity of our findings as the causal effect of this exposure-outcome pair is also statistically significant based on MR-Egger regression, which in design is robust to the directional pleiotropy, and its estimate is consistent with our findings in the main analysis.

Healthspan is defined as the age period free of major age-related morbidities. In the healthspan GWAS study, the top seven age-related morbidities were included (see “Methods”)^13^. In our analysis, healthspan did not show a significant effect on COVID-19-related traits (Fig. 1a). This is unlikely to be due to the power of healthspan GWAS since there were 17 near-independent genome-wide significant SNPs (*P* < 5 × 10^–8^), which is more than in lifespan and longevity GWAS datasets. We, therefore, performed an additional MR analysis to evaluate the role of age-related diseases in age-related COVID-19 risk. The loci for AD, CVD, T2D, cancer, and smoking (or lung cancer) explained the most genetic variance of lifespan, as reported by Timmer et al.^11^. To investigate whether these risk factors contribute to the plausible causal association between lifespan and COVID-19, we conducted an MR analysis of late-onset AD, CAD, T2D, and smoking (the number of cigarettes smoked per day) as exposures (Fig. S2A and Supplementary Data 2). The late-onset AD and CAD were found to significantly increase the risk of COVID-19 infection and hospitalization, while smoking also increases the risk of hospitalization, suggesting the benefit of a longer lifespan on the risk of COVID-19 may be partially mediated by less severe or later occurring of age-related critical disease.

The three lifespan-related traits (lifespan, healthspan, and longevity) are very highly correlated, but each of them is slightly different from the others. For example, the longevity GWAS mainly captured the genetic effect on late-life mortality; lifespan GWAS also includes the genetic effect on early- and mid-life mortality, while the healthspan GWAS additionally represents the disease status of the subjects. As the combined effect of these three traits (healthy aging) is protective against COVID-19 risk (Fig. 1a), we further sought to investigate whether there are marginal effects contributed by the signals specific to individual traits. To do this, we adjusted each of the three lifespan-related traits based on the other two traits, using mtCOJO (see “Methods”). As the exposures are highly correlated, the multivariate MR will have lower power. We had to lower the *P*-value threshold for genetic instruments to 5e−6 to obtain a sufficient number of SNPs for MR analysis. We observed similar univariate estimates for lifespan and longevity as the main analysis after lowering the *P*-value threshold (Fig. S2B). Interestingly, we observed significant protective effects of longer healthspan on COVID-19 infection (OR = 0.90, 95% CI: 0.83−0.97) and hospitalization (OR = 0.85, 95% CI: 0.74−0.99). This gain of power can be explained by the trade-off between the increased number of instruments and the inclusion of more weak instruments.

The effects of each trait on COVID-19 hospitalization remain to be significant after conditioned based on the other two traits. They are largely consistent with univariable analysis, suggesting the existence of marginal effects of each trait on COVID-19 hospitalization that are independent of other traits (Fig. S2B). However, none of the three traits showed protective effects on COVID-19 infection. This result suggests that the protective effect of lifespan-related traits is mainly due to the shared components of the three traits, which are eliminated in the conditional analysis.

Using a similar approach, we further included four lifespan-related risk factors (AD, CAD, smoking, and T2D). After being conditioned on the lifespan-related traits and other three risk factors, the effect of AD on COVID-19 risk is completely removed, with only one nominal significant association showing that the conditioned AD is protective against COVID-19 (Fig. S2C). Similarly, the conditional effect of CAD on COVID-19 risk also became non-significant. These results imply that the effects of AD and CAD on COVID-19 risk are mostly dependent on the effects that are shared with lifespan-related traits instead of the disease itself. Only smoking shows a significant marginal effect on COVID-19 hospitalization, with a consistent estimate as in unconditional analysis, suggesting that the marginal effect of smoking is independent of the other risk factors and lifespan-related traits (Fig. S2C).

### Phenotypic analysis of the association between biological age acceleration and COVID-19 risk

**Fig. 2:**
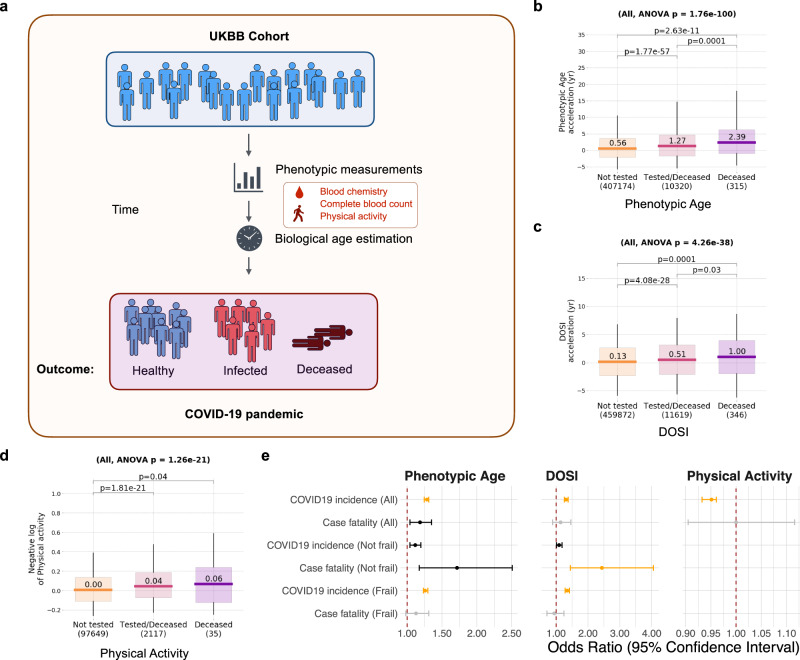
Analysis of association of biological age acceleration with the risk of COVID-19. **a** Schematic representation of analysis of the biological age acceleration in the UKBB cohort. The box plots showing the distribution of biological age acceleration measured by phenotypic age (**b**), DOSI (**c**), and negative log physical activity (**d**) in UKBB subjects that were not tested, tested, or died with COVID-19 infection. Boxes indicate 25−75% interquartile ranges, and whiskers indicate minimum to maximum. The sample size (*N*) for each group is shown under the *x*-axis. **e** The forest plot is showing the predicted effect of biological age acceleration on the risk of COVID-19 in different categories. Error bars show the 95% confidential interval. Significant effects (*P* < 0.001) are in orange. Nominally significant effects (*P* < 0.05) are in black. The odds ratio for Phenotypic Age and Dynamic Organism State Index (DOSI) is given per 10-yr biological age acceleration. The odds ratio for physical activity is given per increase of 1000 steps/day. UKBB UK Biobank, DOSI Dynamic Organism State Indicator.

Therefore, we hypothesized that the strong protective effect of longevity against COVID-19 might not be primarily explained by the age-related morbidities but rather by decelerated biological aging that results in an extended lifespan. To address this hypothesis, we assessed in parallel the three different risk-based biological age predictions computed for the subjects in the UKBB cohort using blood biochemistry (Phenotypic Age), Complete Blood Counts (DOSI), and physical activity measurements^39–41^ (Fig. 2a). We found that COVID-19 incidence in all UKBB datasets was significantly associated with the acceleration of Phenotypic Age, DOSI, and decreased physical activity (Fig. 2b−e, Table 2, and Supplementary Data 3). The estimated odds ratio of COVID-19 infection is 1.28 (95% CI: 1.25−1.31; *P* = 8.4 × 10^–82^) and 1.31 (95% CI: 1.26−1.38; *P* = 9.5 × 10^–32^) for every ten years higher biological age measured by Phenotypic Age and DOSI, respectively. Phenotypic Age and DOSI were also significantly associated with COVID-19 incidence and case fatality independent of the biological age acceleration association with chronic diseases, i.e., separately in cohorts of UKBB individuals having (Frail) or not (Not frail) chronic age-related health conditions (Fig. 2e and Table 2). To assess the causality of this observation, we performed an additional MR analysis to estimate the causal effect of genetically proxied physical activity and epigenetic age acceleration on the risk of COVID-19^20^. Although none of the epigenetic age traits were shown to have a significant effect on COVID-19 after accounting for false discovery rate, the higher walk pace was found to be significantly protective against COVID-19 infection and hospitalization, while a sedentary lifestyle increased COVID-19 susceptibility (Fig. S8). This finding suggests that the association between physical activity and COVID-19 risk observed in the UKBB cohort is likely to be causal.

**Table 2:** Association between biological age acceleration and the risk of COVID-19.

Biological age measurement	Outcome	OR	95% CI	*P*
Phenotypic age	COVID19 incidence (All)	1.28	1.25−1.31	8.4e−82
Phenotypic age	Case fatality (All)	1.19	1.04−1.35	1.1e−02
Phenotypic age	COVID19 incidence (Not frail)	1.12	1.04−1.2	1.9e−03
Phenotypic age	Case fatality (Not frail)	1.72	1.17−2.51	5.4e−03
Phenotypic age	COVID19 incidence (Frail)	1.26	1.23−1.3	3.7e−62
DOSI	COVID19 incidence (All)	1.31	1.26−1.38	9.5e−32
DOSI	COVID19 incidence (Not frail)	1.09	1.01−1.19	3.6e−02
DOSI	Case fatality (Not frail)	2.44	1.45−4.06	7.7e−04
DOSI	COVID19 incidence (Frail)	1.35	1.28−1.42	3.6e−28
Physical activity	COVID19 incidence (All)	0.95	0.93−0.96	9.1e−19

Only the associations that reached nominal significance (*P* < 0.05) are shown.

We also observed elevated biological age acceleration of all measures of biological age (Fig. 2b−d, Fig. S3A−D, and Supplementary Data 3) in cohorts of individuals who died from COVID-19 compared to those tested (and most probably suffering from the disease), and, separately, in cohorts of those tested versus the rest of UKBB (and presumed free of the disease). The number of UKBB subjects with data fields required for the Phenotypic Age and DOSI was comparable, and we found that Phenotypic Age comparisons produced a better statistical power. The number of UKBB subjects with physical activity metrics was small, but the association of biological age acceleration in the form of physical activity deficit and the incidence of COVID-19 was significant.

### Bivariate genomic scan of aging-related COVID-19 risk

**Fig. 3:**
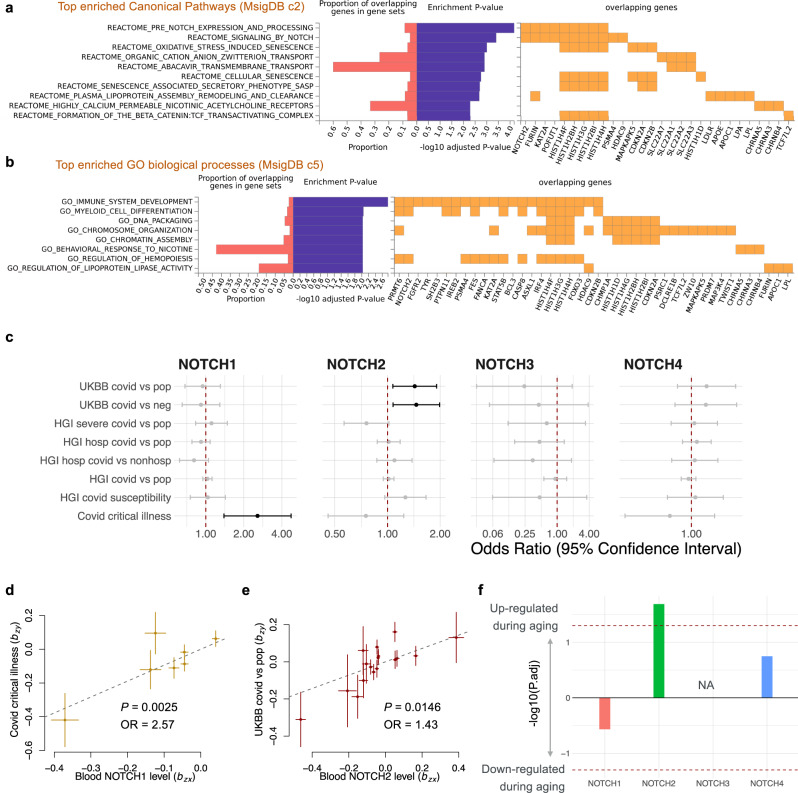
Bivariate genomic scan identified Notch signaling as an age-related COVID-19 risk. Gene set enrichment analysis of aging-related COVID-19. Top significantly enriched (*P*_adjusted_ < 0.05) canonical pathways (**a**) and GO biological processes (**b**) are shown. **c** Forest plot showing Mendelian randomization estimates for the causal effect of blood *NOTCH2* expression on the risk of COVID-19 infection. Error bars show the 95% confidential interval. Nominally significant effects (*P* < 0.05) are in black. Error bars show the 95% confidential interval. **d, e** Plots of effect size of all genetic instruments from blood eQTLs for *NOTCH1* versus those for COVID-19 critical illness (**d**) and *NOTCH2* versus UKBB COVID-19 infection (**e**). Error bars represent standard errors. **f** Bar plot showing the age-related differential expression of *NOTCH1-4* in blood. The *y*-axis represents the −log10(P.adj) × sign of changing direction, i.e., the positive value represents an age-related increase. UKBB UK Biobank, HGI Host Genetics Initiative.

To gain mechanistic insights into how aging and COVID-19 intertwined at the genetic level, we performed a bivariate genomic scan using the GWAS of healthy aging and UKBB COVID-19 infection to identify the genetic variants that contribute to both aging and the risk of COVID-19, i.e., aging-related COVID-19 risk (Fig. S4, see “Methods”). We identified twenty bivariate loci at genome-wide significance (*P* < 5 × 10^−8^), where the null hypothesis is no association with healthy aging and COVID-19 infection (Fig. S4). The summary statistics of aging-related COVID-19 risk were then annotated using FUMA and a functional enrichment analysis in 2868 canonical pathways (including gene sets from BIOCARTA, KEGG, PID, REACTOME, and WikiPathways) and 7350 Gene Ontology (GO) biological processes was performed. We found significant enrichment (*P*_adjusted_ < 0.05) in 67 canonical pathways and 26 biological processes. The canonical pathways with the strongest enrichment included pre-Notch expression and processing (*P* = 3.0 × 10^−8^), signaling by Notch (*P* = 3.6 × 10^−7^), and oxidative stress-induced senescence (*P* = 1.4 × 10^−6^) (Fig. 3a and Supplementary Data 4). Top enriched biological processes were immune system development (*P* = 2.3 × 10^−7^) and myeloid cell differentiation (*P* = 2.4 × 10^−6^), among others (Fig. 3b and Supplementary Data 4).

### Association between NOTCH signaling and aging-related COVID-19 risk

The Notch pathway is an evolutionally conserved signaling pathway, which is thought to be involved in both age-related inflammation and the development of age-related disease^42^. Moreover, Notch signaling is related to the entry of SARS-CoV-2 through the positive regulation of host proteins that promote the entrance of the virus into the cell (e.g., FURIN and ACE2)^43^. In humans, there are four paralogs in the Notch family (*NOTCH1-4*)^44^. We hypothesized that Notch signaling is a mediator of aging-related COVID-19 infection, and its effect may be related to the expression of *NOTCH*. This hypothesis was investigated with MR of blood eQTLs of *NOTCH1-4* from eQTLgen^26^, against COVID-19-related traits (Fig. 3c and Table 3). We found that per standard deviation, higher expression of *NOTCH1* in whole blood increases the risk of critical illness of COVID-19 by 157% (Fig. 3d, OR 95% CI: 1.39−4.74, *P* = 0.0025), and higher expression of *NOTCH2* in whole blood increases the risk of COVID-19 infection by 43% (Fig. 3e, OR 95% CI: 1.39−4.74, *P* = 0.0025). We also observed a similar odds ratio estimate with overlapping 95% CI using the maximum likelihood method, MR-Egger method, and simple median method, but with a wider 95% CI that includes null in the sensitivity analysis for *NOTCH1-2*. The MR-Egger intercept term was not deviated from zero for *NOTCH2*, suggesting there is no imbalanced pleiotropic effect in eQTLs (Table S3). Note that due to the limited number of available eQTLs, we could not perform MR-Egger and simple median method on *NOTCH1* and critical illness of COVID-19 (Fig. S5).

**Table 3:** Mendelian randomization estimates for the association between *NOTCH2* expression and risk of COVID-19.

Exposure	Outcome	OR	95% CI	*P*
*NOTCH1*	Covid critical illness	2.57	1.39−4.74	0.0025
*NOTCH2*	UKBB covid vs. neg	1.46	1.08−1.99	0.0150
*NOTCH2*	UKBB covid vs. pop	1.43	1.07−1.91	0.0150

Only the associations that reached nominal significance (*P* < 0.05) are shown.

To further explore the tissue-specific effect of *NOTCH1-4* expression on COVID-19, we performed an MR analysis using the tissue eQTL from GTEx V8 (Fig. S6). Due to the limited sample size in GTEx, there were no or only a few significant Notch eQTLs in most tissues, especially for *NOTCH1-3*. Among the testable tissues, we found that *NOTCH2* expression in the colon and esophagus increased the risk of COVID-19, with larger effect sizes and significance compared with the effect estimate from blood eQTL (Fig. S6). In addition, although we didn’t observe the risk associated with *NOTCH3* and *NOTCH4* expression in whole blood, *NOTCH3* expression in the lung and thyroid, as well as *NOTCH4* expression in the brain increased the risk of COVID-19 infection (Fig. S6). These results suggest a causal role for the Notch family, and more generally, Notch signaling, in the risk of COVID-19. We further examined the dataset of Peters et al.^27^, which contains associations of genes with age in humans, estimated from 7,074 whole blood samples. Among *NOTCH1-4*, only *NOTCH2* significantly (*P* = 0.007) increased during aging, suggesting that the age-related increase of COVID-19 risk may partially be mediated through the increase in *NOTCH2* expression. Additional mechanistic work would be necessary to provide further evidence for a causal link between Notch, aging, and COVID-19.

### Effects of 389 immune cell traits on aging and COVID-19

**Fig. 4:**
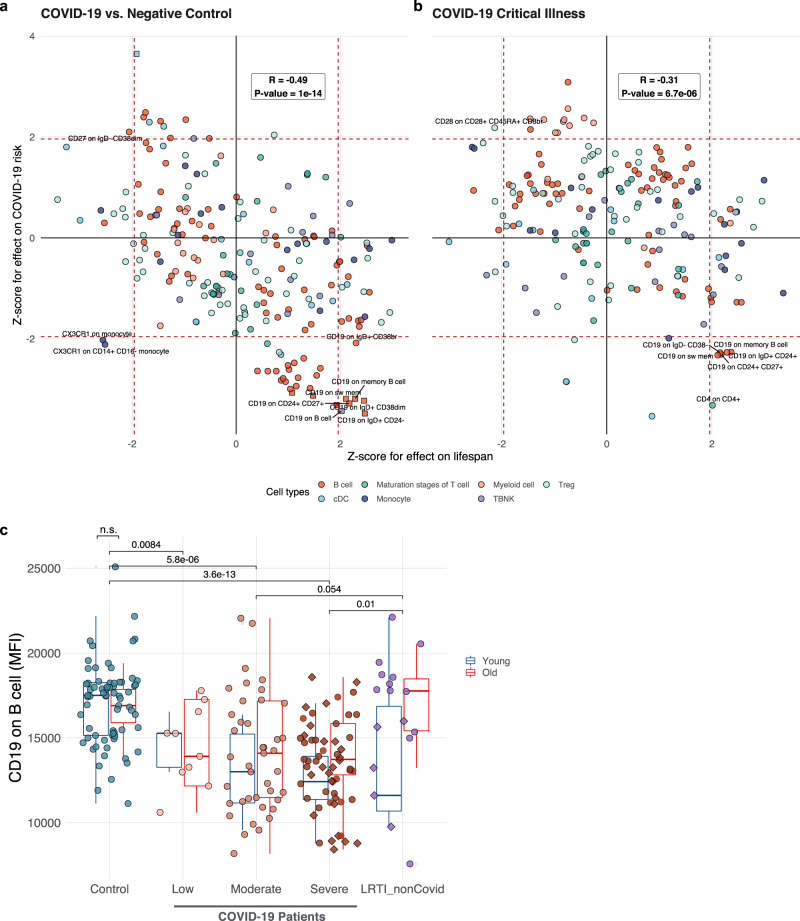
Mendelian randomization on 389 immune cell-type-specific surface markers and analysis of clinical data reveal the role of CD19 in B cells. Mendelian randomization results showing the effect of immune cell surface marker levels on lifespan (*x*-axis), and the *y*-axis shows the *Z*-score for the risk of COVID-19 infection (*N*_trait_ = 389) (**a**) and COVID-19 critical illness (**b**). Red dashed lines to denote the ±1.96 *Z*-score threshold (equivalent to *P* < 0.05). Only traits with *P* < 0.05 for both outcomes are annotated. Traits with FDR < 0.05 for at least one outcome are shown in squares. **c** Box plot showing the CD19 level in B cells in different groups (*N*_control_ = 70, *N*_low_ = 10, *N*_moderate_ = 41, *N*_severe_ = 58, *N*_LRT_ = 17). Blue boxes represent younger subjects (age < 60) and the red boxes older subjects (age > 60). Subjects that have been admitted to ICU are in the diamond. The center line in the box shows the median. The bottom and top of the box show the 25th and 75th quantiles. The whiskers represent the expected variation of the data. The whiskers extend 1.5 times the IQR from the top and bottom of the box. cDC classical dendritic cells, TBNK T cells B cells and natural killer cells, MFI median fluorescence intensities, LRT lower respiratory tract infections.

Regulation of immune cell development is a major function of Notch signaling^45^. Interestingly, the top enriched GO term for aging-related COVID-19 risk is immune system development (Fig. 3b). Surface antigens (e.g., cluster of differentiation (CD) molecules) expressed in immune cells play critical roles in immune cell function and are essential markers for immune cell types^46^. To gain further insight into how the immune system affects aging-related COVID-19 risk, we performed a systematic MR analysis using 389 immune cell-type-specific surface markers represented by MFIs as exposure^28^, and explored their effects on lifespan and COVID-19 risk. Specifically, we considered two components of COVID-19 risk: the risk of infection, represented by COVID-19 cases versus negative controls (Fig. 4a), and the risk of developing severe symptoms after infection, represented by COVID-19 cases with critical illness (admission to ICU) (Fig. 4b). We focused on 243 MFI traits whose causal effect can be estimated for both lifespan and COVID-19 related traits. Among these traits, we observed significant negative correlations between their effects on lifespan and COVID-19 risk, both for the risk of infection (Pearson’s *r* = −0.49, *P* = 1e−14) and the risk of developing critical illness (Pearson’s *r* = −0.31, *P* = 6.7e−6), indicating that the immune-related traits that lead to a longer lifespan also tend to decrease COVID-19 risk in both categories, and *vice versa*. We then examined the correlation in individual cell types. B cell-related traits showed the strongest negative correlation of the effect on lifespan and COVID-19 risk (Fig. S7) in terms of infections (*r* = −0.71) and severity (*r* = −0.48). This finding is consistent with our results (Fig. 1), suggesting that the immune function, especially B cells, at least in part mediates the effect of aging on COVID-19 risk.

To identify individual traits important for both lifespan and COVID-19 risk, we assessed the candidate traits that reach nominal significance threshold (*P* < 0.05) for both lifespan and COVID-19 risk, and also with FDR < 0.05 for at least one of the outcome traits. Only six traits satisfy these criteria. Interestingly, all of them represent the CD19 levels in different subsets of B cells, suggesting that higher CD19 levels in B cells lead to a longer lifespan and lower risk of COVID-19 infection (Fig. 4a and Supplementary Data 5). Four of these six traits also reached nominal significance for their effect on the risk of developing critical illness (Fig. 4b and Supplementary Data 5), suggesting that higher CD19 in B cells may reduce COVID-19 severity.

To validate the clinical relevance of our finding, we analyzed a clinical dataset from the COVID-IP (Covid–ImmunoPhenotype) project (Fig. 4c), which provides the MFIs measurement of CD19 in B cells in COVID-19 patients, healthy controls, and patients with non-COVID-19 lower respiratory tract infections (LRTIs)^47^. Consistent with the estimation of MR, healthy subjects showed a significantly higher expression of CD19 in B cells, compared to COVID-19 patients with low, moderate, and severe symptoms (Fig. 4c and Supplementary Data 6). CD19 is a member of the immunoglobulin superfamily expressed exclusively in B cells, and it facilitates their activation^48^. Although we did not observe significant changes in CD19 levels between young and old subjects in any of the groups, previous studies suggest that the number of CD19 + B cells decreases during aging^49^, especially in men who are more susceptible to COVID-19^50^. B cells are severely depleted in COVID-19 patients and fail to form germinal centers^51–53^, thus providing a link between proper B-cell development and COVID-19. In addition, activation of Notch signaling was shown to interfere with the development of B cells and decreased CD19 levels, which makes further connections to our findings involving the NOTCH gene family (Fig. 3)^54^. Since successful elimination of respiratory infections is dependent on B cell activation through CD19^55^, and respiratory infections are one of the leading causes of death in the elderly, our findings may explain the apparent genetic link between lifespan and COVID-19 through CD19 expression.

### Genetic correlations across lifespan-related traits, age-related diseases, and COVID-19 risk

**Fig. 5:**
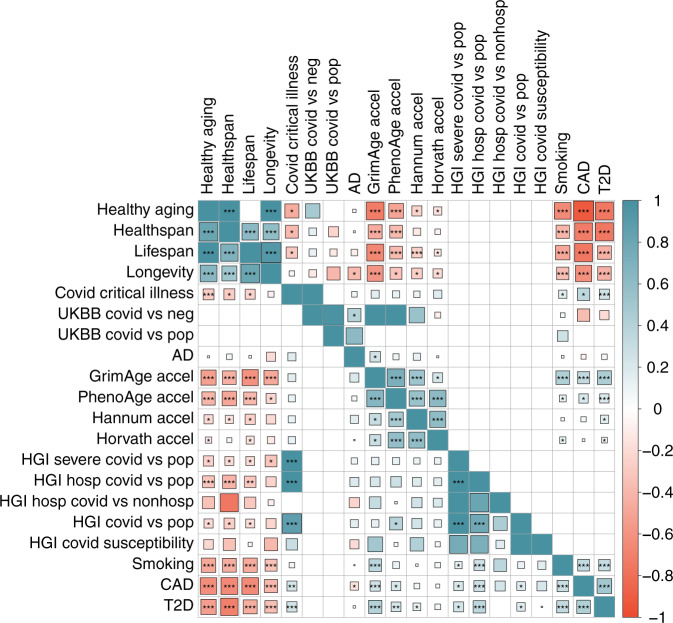
Genetic correlation estimates from HDL and LDSC among phenotypes. Upper triangle: HDL estimates; lower triangle: LDSC estimate. The areas of the squares represent the absolute value of corresponding genetic correlations. The genetic correlation that could not be estimated is blank. *P* values are corrected using Bonferroni correction for 190 tests, *P_nominal_ < 0.05, **P_adjusted_ < 0.05, ***P_adjusted_ < 0.01. AD Alzheimer’s disease, accel acceleration, CAD coronary artery disease, T2D type-II diabetes.

**Fig. 6:**
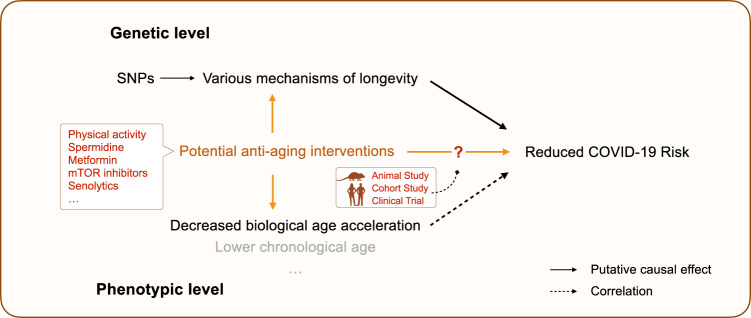
Schematic diagram showing the link between aging and COVID-19. Black solid arrows show the causal effect established by Mendelian Randomization analysis, and the black dashed arrows show the correlation observed in the UKBB cohort. Orange arrows show potential approaches to reduce COVID-19 risk. SNPs single nucleotide polymorphisms.

Next, we estimated genetic correlations across all traits using LD score regression and HDL methods (Fig. 5 and Supplementary Data 7)^37,38^. After adjusting for 190 tests (FDR < 0.05), we observed four pairs of significant genetic correlations between four lifespan-related traits and eight COVID-19 traits. COVID-19 hospitalization (with population control) negatively correlated with healthy aging (rg = −0.33, *P* = 5.8e−6), healthspan (rg = −0.42, *P* = 7.1e−6), and lifespan (rg = −0.30, *P* = 2.0e−4). Critical illness of COVID-19 also negatively correlated with healthy aging (rg = −0.27, *P* = 2.1e−6). The rest of 28 pairs of lifespan-related traits and COVID-19 traits were, although less significantly, negatively correlated with each other, with nine pairs with *P* < 0.05 (Fig. 5). In addition, we observed a positive genetic correlation between PhenoAge acceleration and risk of COVID-19 infection (rg = 0.38, *P* = 0.01). Together, these findings are consistent with our hypothesis and support a shared genetic mechanism between aging and COVID-19 risk (Fig. 6 and Supplementary Data 8).

Finally, to evaluate other risk factors for COVID-19 infection and severity, we conducted a separate MR analysis using GWAS data of 22 common diseases from GERA^23^ (Fig. S9 and Table S4) but did not find a significant association based on FDR. Among the nominally significant associations, cancer and dyslipidemia increased the risk of COVID-19 infection, and Hypertensive disease increased the risk of COVID-19 with respiratory failure while decreasing the risk of infection. A phenome-wide association analysis using a more powerful disease GWAS dataset in the future might provide a complete picture of how common diseases affect COVID-19 risk.

## Discussion

In this study, we explored a potential causal relationship between aging and the risk of COVID-19 by conducting a multi-instrument MR analysis using four different lifespan-related traits as exposures and various COVID-19-related traits as outcomes. We found that genetically proxied longer lifespan and longevity were significantly associated with the decreased risk of COVID-19, and further analyses revealed a key role of elevated biological age and severity of chronic age-related diseases in this association. One of the key contributing factors in these associations was found to be the immune response. The competence of the immune system declines as people age, which is known as “immunosenescence”^56^. The hallmarks of immunosenescence include the impaired response to new antigens, decreased receptor diversity, and chronic inflammation. As a result, elderly subjects are more susceptible to infectious diseases, including COVID-19, and have a poor response to vaccines^56,57^. On the other hand, it has been reported that circulating immune cells in centenarians possess unique characteristics that sustain immune responses to infections^58^. Moreover, the offspring of centenarians were shown to have a lower level of pro-inflammatory cytokines and better hematopoiesis^59^, suggesting that the benefits on the immune system in centenarians are heritable. Therefore, a better immunological profile in people with pro-longevity genetics may support the observed effect of longevity on COVID-19.

The Notch pathway is an evolutionally conserved signaling pathway involved in age-related inflammation and diseases^42^. Notch signaling is related to the entry of SARS-CoV-2 through the positive regulation of host proteins that promote the entrance of the virus into the cell^43^, which is mediated by the binding of viral S (spike) glycoprotein to the Angiotensin-Converting Enzyme 2 (ACE2)^60^. Therefore, upregulation of ACE2 could potentially increase the risk of viral infection. ADAM17 (A Disintegrin And Metalloproteases 17) is a metalloprotease that supports the shedding of ACE2 on the cell membrane^61^. It is negatively regulated by Notch signaling, whereas downregulation of ADAM17 significantly reduces the ACE2 shedding^43^. Besides ADAM17, a proteolytic cut of the S protein mediated by furin after S glycoprotein binds to ACE2 is required for the entry of SARS-CoV-2 into the cell. Furin expression is positively regulated by Notch signaling, and furin is also involved in the maturation of Notch precursor^43^. All this evidence is in line with our finding that Notch signaling plays an important role in aging-related COVID-19.

*NOTCH2* is one of the four Notch paralogs in mammals. Our MR analysis revealed a potential causal relationship between *NOTCH2* expression and COVID-19 infection (Fig. 3e). A previous study suggested that *NOTCH2* promotes goblet cell metaplasia in the lung, which is the hallmark of airway diseases^62^. Moreover, goblet cells are the major source of ACE2 in the lung, playing a role in enabling COVID-19 infection. Therefore, increased *NOTCH2* expression during aging may play a causal role in the increased risk of COVID-19 infection in the elderly. We observed a relatively large effect size (43% increased risk of infection for every one standard deviation higher *NOTCH2* expression), suggesting that *NOTCH2* may be a desirable target in COVID-19, as well as a marker of a population with a higher potential risk of infection. Besides *NOTCH2*, we found that the expression of the other three paralogs also increases the risk of COVID-19 in a tissue-specific manner. Notably, *NOTCH4* was identified as the leading genetic risk locus for the critical illness of COVID-19^24^. However, the NOTCH4 locus is located in the major histocompatibility complex region^24^, and is not replicated in other cohorts. Therefore, further experimental and clinical studies are needed to validate the causal relationship between the Notch family and age-related COVID-19 risk.

Aging manifests as progressive systemic remodeling of the organism, and hence a great number of biological measurements are associated with age. Several sets of physiological and biological indices have been proposed for quantification of aging progression in the form of biological age^63,64^ and frailty index^65,66^. One popular approach is to regress relevant variables to predict chronological age and thus produce the “biological age” prediction. Popular Horvath, Hannum, and other methylation age-clock models, as well as other clocks, are the widely used examples of such an approach^67,68^.

An interesting alternative is to produce the log-linear all-cause mortality estimate with a proportional hazard model and treat the resulting value as a measure of biological age. Phenotypic Age from blood biochemistry markers^39^, DOSI from CBC^40^, averaged physical activity levels^41^, and more sophisticated machine learning algorithms used to predict the risk of death from physical activity time series of wearable devices^69^, or even self-reported health questionnaires, are all examples of this approach^70^. All reliable biological age predictors are associated with chronic disease burden, unhealthy lifestyles such as smoking (both overall and in disease-free population), and future incidence of chronic diseases in healthy subjects^39–41,63,64,66,71,72^. In our work, we established the association of biological age acceleration with the risk of non-chronic disease, COVID-19, and the corresponding case fatality in the UKBB cohort independent of disease burden. The association was significant for biological age acceleration measures obtained from blood biochemistry (Phenotypic Age)^39^, CBC (DOSI)^40^, and mean physical activity (number of steps per day recorded by wearable devices over a week-long period of time^41^; the number of UKBB subjects with physical activity measurements was too low for separate biological age acceleration characterization in frail and non-frail cohorts).

Decreased physical activity was associated with an increased risk of COVID-19 in the UKBB cohort. This observation may be interesting on its own since the widespread lockdown measures brought about a dramatic (up to 27.3%, which is 1,432 steps per day, within 30 days) decline of average physical activity^73^. Our association study suggests a more than 10% risk increase corresponding to 1.5 thousand steps per day loss. There are feedback loop effects of decreased mobility on biological age acceleration measures, and as such, the associated risk adjustments must be taken into account in advanced epidemiological models of lockdown effects. Yet, it was not clear whether this is an effect of chronic diseases, also negatively affecting mobility. A biological age model built from consumer-grade wearable sensors data, the GeroSense biological age acceleration, was better associated with the incidence of COVID-19 than the average physical activity level in UKB. The result persisted among a sub-population of individuals free of chronic health conditions^70^.

One advantage of our study design is that all the biological age acceleration predictors were measured prior to the pandemic. Therefore, the association between biological age acceleration and the risk of COVID-19 (and probably other dangerous infectious diseases) is free of reverse causation and likely to be causal if there are no other confounders. Thus, our research supports the idea that the pro-active application of anti-aging (that is, biological age-reducing) drugs in a prophylaxis mode may protect biomarker-defined vulnerable individuals. And, reversely, a significant reduction of biological age by an experimental drug in a clinical trial (probably as early as phase I) could warrant further clinical studies in elderly subjects.

The association of biological age acceleration with case fatality was weaker (only Phenotypic Age acceleration exhibited a significant effect). This can be explained by the considerably smaller number of UKBB subjects involved in the statistical analysis (346 dead individuals compared to 11,619 tested (and presumed sick) and 459,872 overall subjects in UKBB). The case fatality rate increases exponentially with age, and therefore it would be reasonable to expect the association of biological age acceleration with the risk of death in COVID-19 patients. We expect future studies to corroborate our findings. Whether or not this association is causative could not be established in our study.

The correlation between COVID-19 risk, lifespan, and immune phenotypes highlighted the importance of B cells and CD19 expression. CD19 is expressed throughout all stages of B-cell development and is critical for humoral responses to infection. B cell numbers decrease in blood with age and with COVID-19^49–53^, providing a link between the two processes. Despite decreasing number of overall B cells, B cells that with lower CD19 expression have more frequently IgM + B cells in severe COVID-19 patients, suggesting that B cells undergo plasmacytoid maturation and immunoglobulin switching due to SARS-CoV-2 infection^51^. However, little is known about the role of CD19 in it and whether the loss of B cells is detrimental or adaptive. It is possible that the genetic predisposition for higher CD19 expression results in easier activation of B cells and improved production of antibodies against virus injection. Future mechanistic studies are needed to address these questions, and in particular, to test whether therapies preserving healthy levels of CD19 in B cells can extend lifespan and protect from COVID-19.

There are multiple clinical trials proposed to employ potential lifespan-extending drugs to protect the elderly from COVID-19, based on promising observational data on metformin^5–9^. However, epidemiological studies are prone to confounding, reverse causation, and various biases, and therefore are an unreliable indicator of the causal associations. MR is a method that utilizes genetic instruments that are robustly associated with exposures and thus generate more reliable evidence in predicting novel interventions^74^. In our MR analysis, we found evidence for the causal relationship between longevity and decreased COVID-19. The analysis of genetic risk factors and phenotypic measurements suggests that this causal effect is likely to be mediated by the decelerated rate of aging, which can be captured by biological age measurements. Therefore, our finding supports a possibility of using lifespan-extending drugs against COVID-19 when one of the following assumptions holds: (1) the selected anti-aging drugs extend lifespan through a mechanism that mimics the genetics of longevity; and (2) the selected anti-aging drugs could slow down or reverse the aging process measured by biological age models (e.g., phenotypic age).

While the first assumption is hard to test, recent studies suggest that some anti-aging interventions can slow down and even reverse the biological age measured by biological age models^75^. For example, a cocktail treatment of recombinant human growth hormone, dehydroepiandrosterone, and metformin reversed the immunosenescent trend, and the biological age was measured by several biological age models (including PhenoAge) was reversed by 2.5 years on average after 12 months of treatment^75^. Thus, it could be worthwhile prioritizing established anti-aging drugs in COVID-19 clinical trials (Fig. 6).

## Supplementary information

Supplementary Material

Supplementary data 1

Supplementary data 2

Supplementary data 3

Supplementary data 4

Supplementary data 5

Supplementary data 6

Supplementary data 7

Supplementary data 8

Reporting Summary

Description of Additional Supplementary Files

## Data Availability

GWAS summary statistics used in this study are publicly available (for URLs, see Table S1). The individual-level phenotype data are available in the UK Biobank (http://www.ukbiobank.ac.uk/) upon application and with permission of UKBB’s Research Ethics Committee. The source data for the main figures can be accessed as Supplementary Data 2−6. The bivariate GWAS summary statistics of aging-related COVID-19 generated in this study are available in Figshare (https://figshare.com/articles/dataset/combined_ukbbCOVID_meta_txt/16416822).

## Code availability

The GSMR analysis was performed using GCTA 1.93.1beta available at https://cnsgenomics.com/software/gcta/. The genetic correlation analysis was performed using LDSC v1.0.1 available at https://github.com/bulik/ldsc, and HDL v1.3.8 available at https://github.com/zhenin/HDL.
