# Supplementary material for: Genetic and phenotypic analysis of the causal relationship between aging and COVID-19
Source: Commun Med (Lond). 2021 Oct 5;1:35. doi: 10.1038/s43856-021-00033-z (PMC9053191; doi:10.1038/s43856-021-00033-z)
Supplement: Supplementary file 1 — Supplementary Material [file 43856_2021_33_MOESM1_ESM.pdf]

Supplementary Tables and Figures

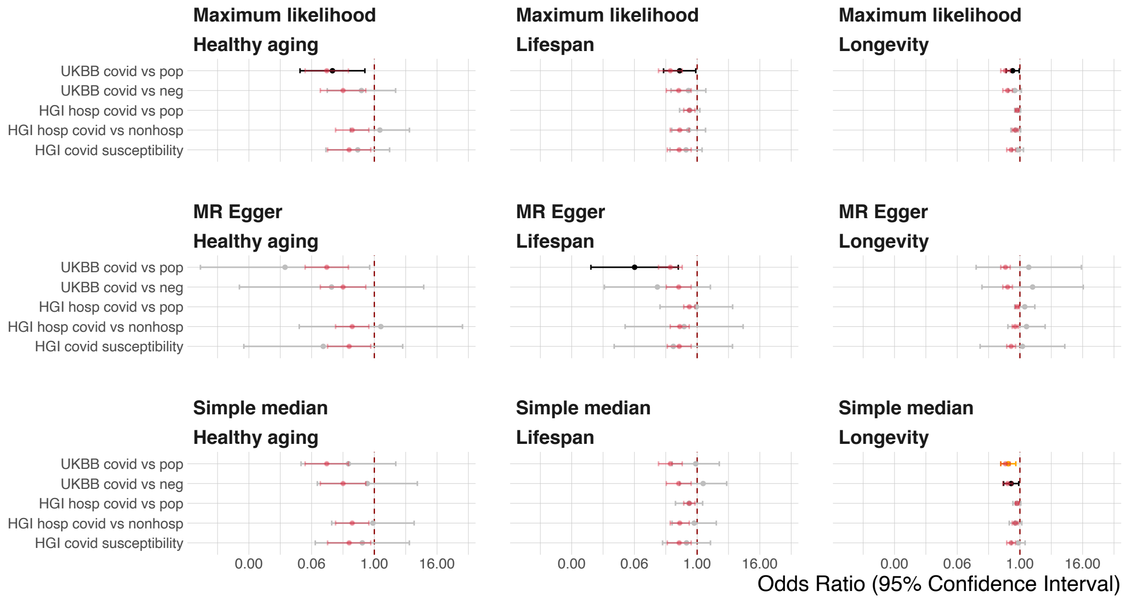

Figure S1. Sensitivity analysis of the association between lifespan-related traits and the risk of COVID-19. This forest plot shows Mendelian randomization estimates for the causal effect of lifespan-related traits on the risk of COVID-19 based on three different MR methods (Maximum likelihood, MR-Egger, and simple median). Error bars show 95% confidential interval. Significant effects with FDR < 0.05 are in orange. Nominally significant effects (*P* < 0.05) are in black. Red error bar represents the estimated causal effect from the main analysis.

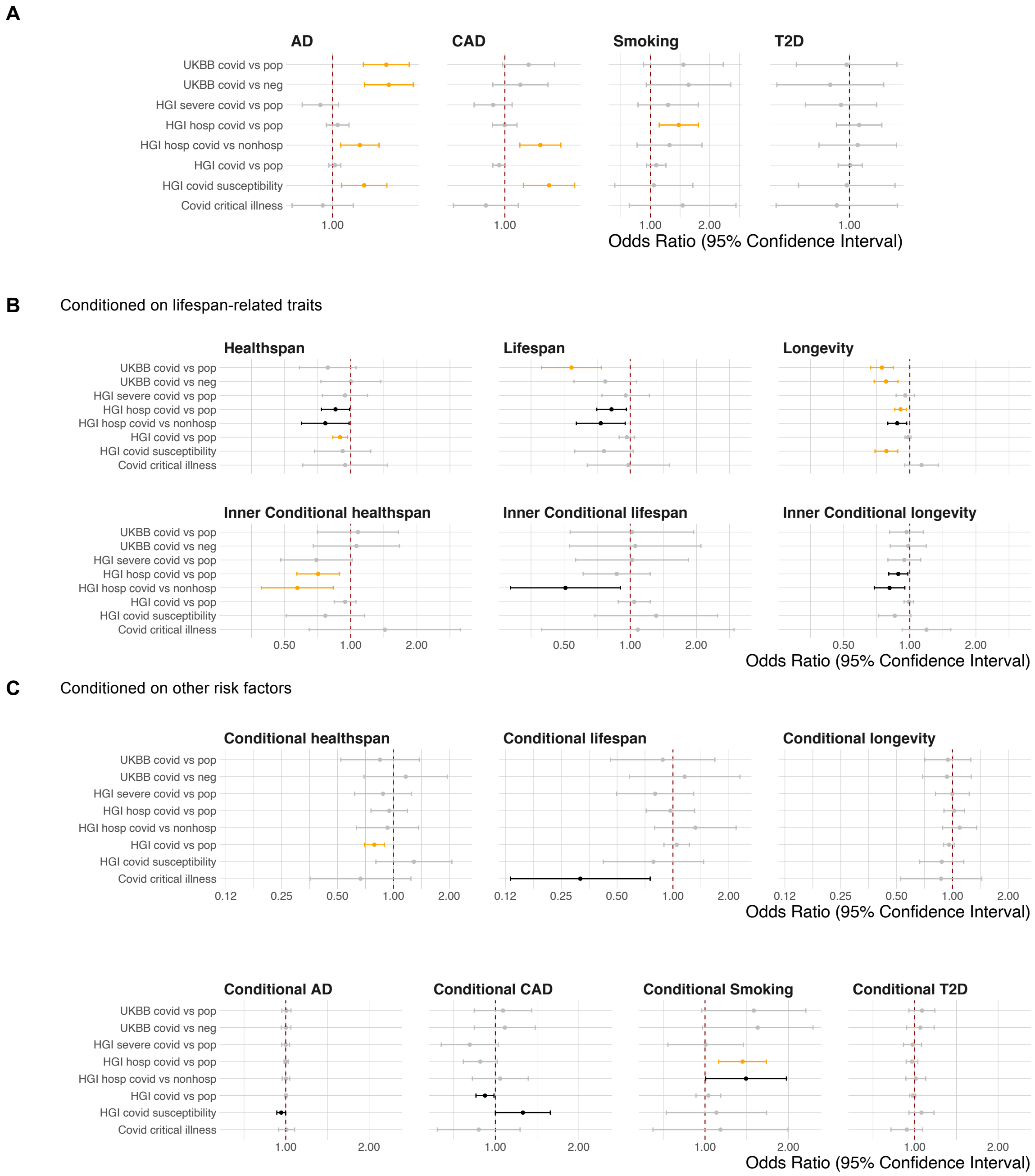

Figure S2. Conditional analysis of the association between lifespan-related traits and the risk of COVID-19. A. Forest plot showing Mendelian randomization estimates for the causal effect of age-related risk factors on the risk of COVID-19. **B.** Forest plot showing Mendelian randomization estimates for the causal effect of univariate and multivariate lifespan-related traits on the risk of COVID-19. **C.** Forest plot showing Mendelian randomization estimates for the causal effect of traits (conditioned on age-relate risk factors) on the risk of COVID-19. Error bars show the 95% confidential interval. Significant effects with FDR < 0.05 are in orange. Nominally significant effects (*P* < 0.05) are in black.

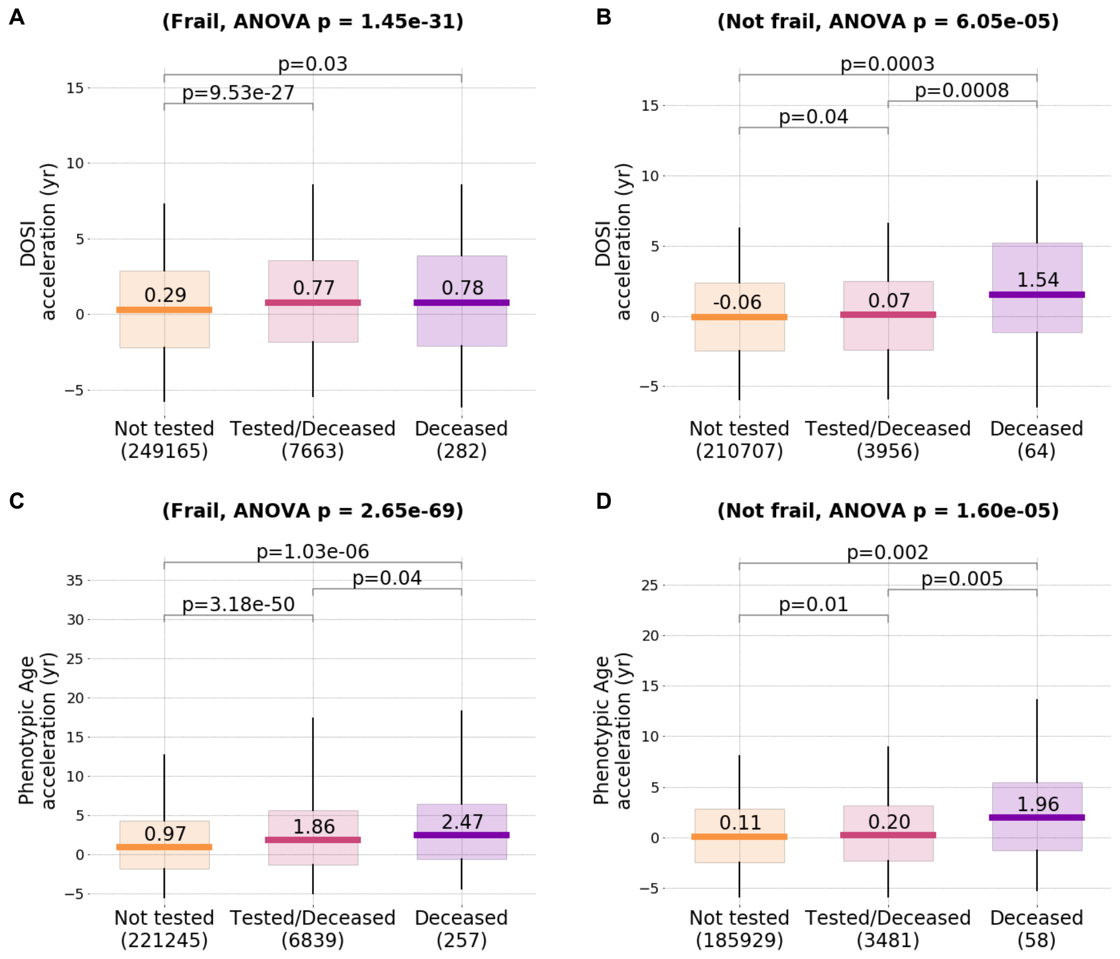

Figure S3. Biological age acceleration in COVID-19 patients from UKBB cohort. Box plot showing the distribution of biological age acceleration measured in different groups. The sample size (N) for each group is shown under the x-axis. Boxes indicate 25%-75% interquartile ranges, and whiskers indicate minimum to maximum. **A, B.** BAA measured by DOSI. **C, D.** BAA measured by Phenotypic Age.

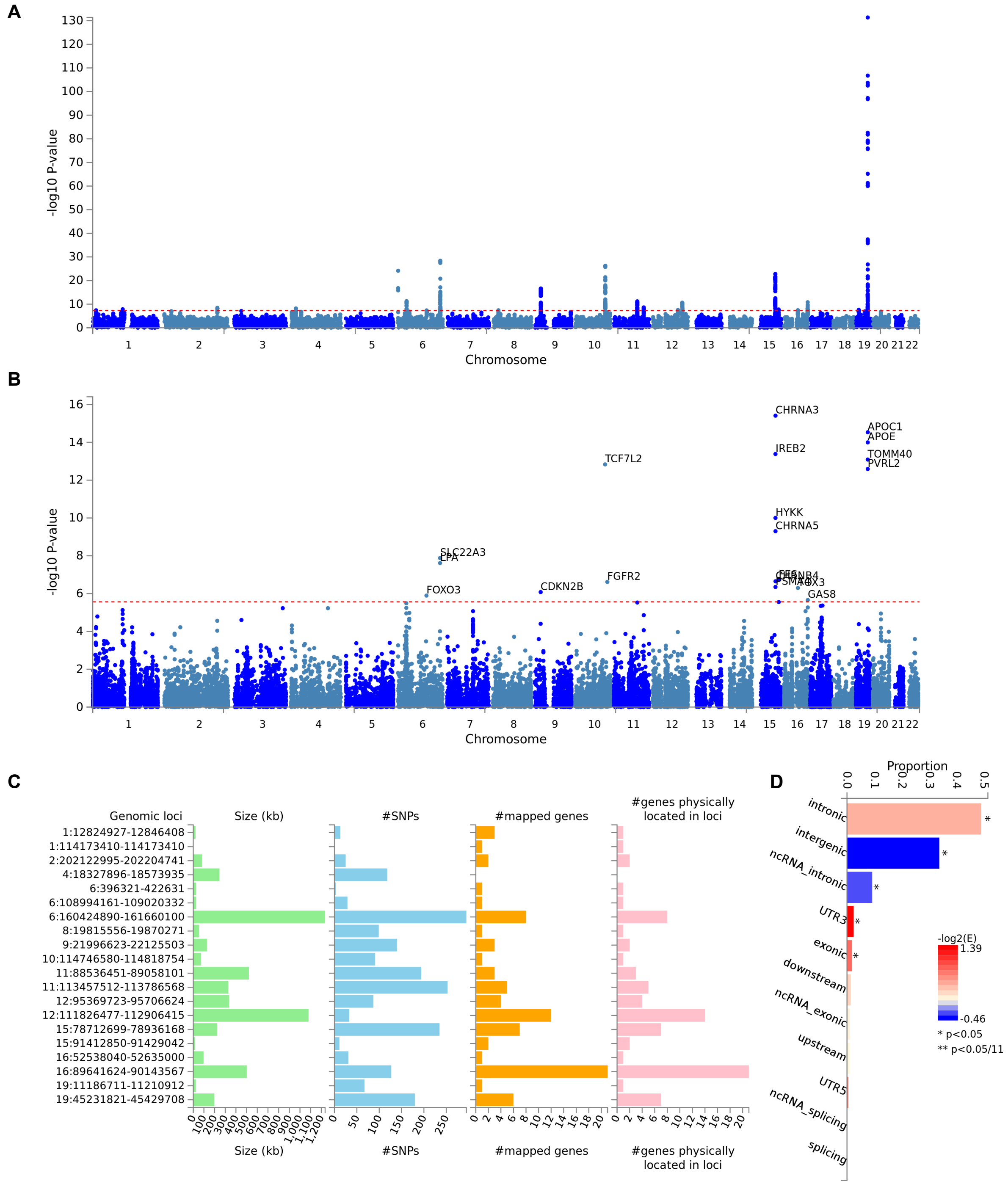

Figure S4. Twenty bivariate loci identified at genome-wide significance. A. Manhattan plot showing the nominal strength of association −log10(P value) on the y-axis against the chromosomal position of SNPs on the x-axis, where the null hypothesis is no association with healthy aging and COVID-19 infection. **B.** Manhattan plot of the gene-based test as computed by MAGMA test. SNPs were mapped to 18370 protein coding genes. Genome wide significance (red dashed line in the plot) was defined at P = 0.05/18370 = 2.722e-6. **C.** The histogram presenting summary results per genomic locus. **D.** Histogram displays the proportion of SNPs with functional annotation assigned by ANNOVAR. Bars are colored by log2(enrichment) relative to all SNPs in the selected reference panel.

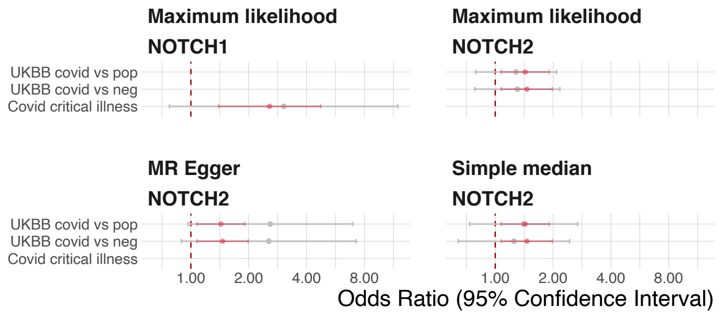

Figure S5. Sensitivity analysis of the association between Notch and the risk of COVID-19. Forest plot showing Mendelian randomization estimates for the causal effect of blood expression of Notch1/2 on the risk of COVID-19 from three different MR methods (Maximum likelihood, MR-Egger, and simple median). Error bars show the 95% confidential interval. Significant effects with FDR < 0.05 are in orange. Nominally significant effects (*P* < 0.05) are in black. Red error bar represents the estimated causal effect from the main analysis.

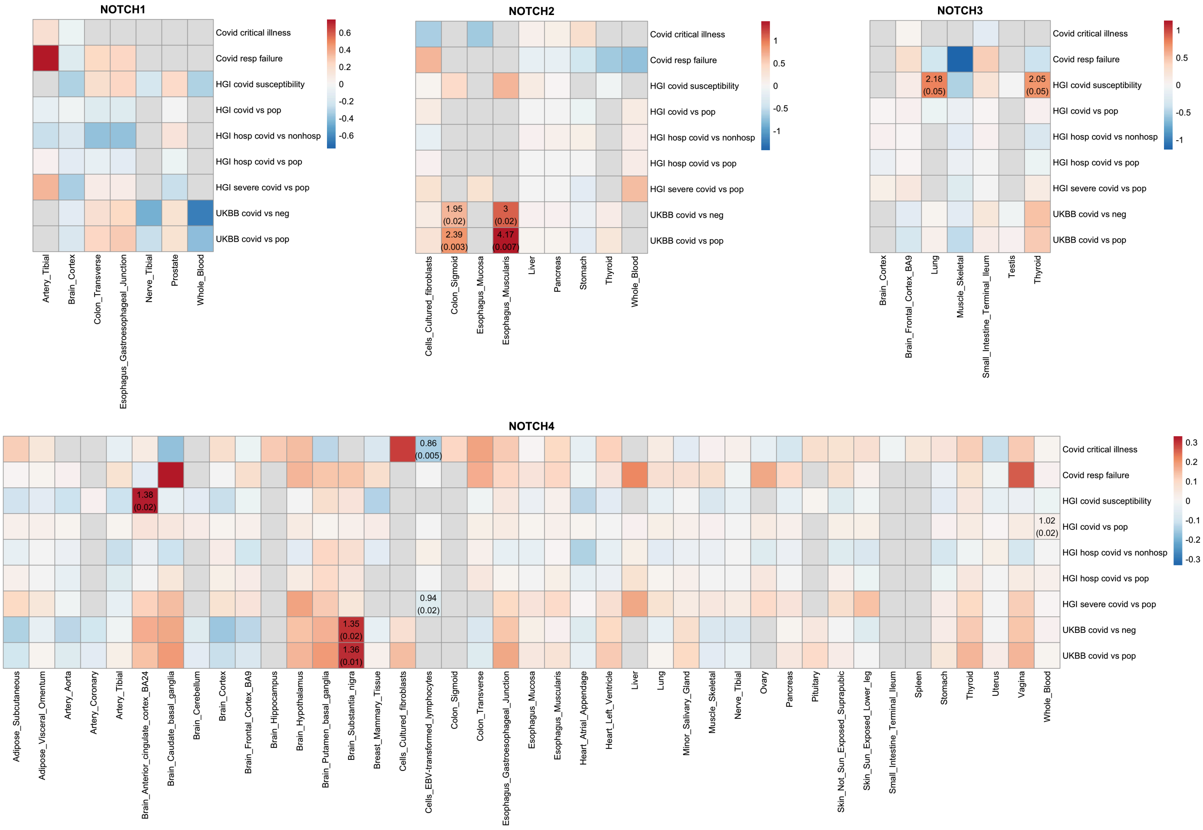

Figure S6. Mendelian randomization analysis investigating the association of tissue-specific Notch expression with the risk of COVID-19. Shown are the results of GSMR analyses with tissue-specific Notch expression from GTEx V8. Colors represent the effect sizes (as measured by log odds ratios, log ORs) of expression on COVID-19, red for risk effects, blue for protective effects, and gray means the effect cannot be estimated due to limited available SNPs. Nominally significant effects ($P < 0.05$) are labeled with OR (P-value).

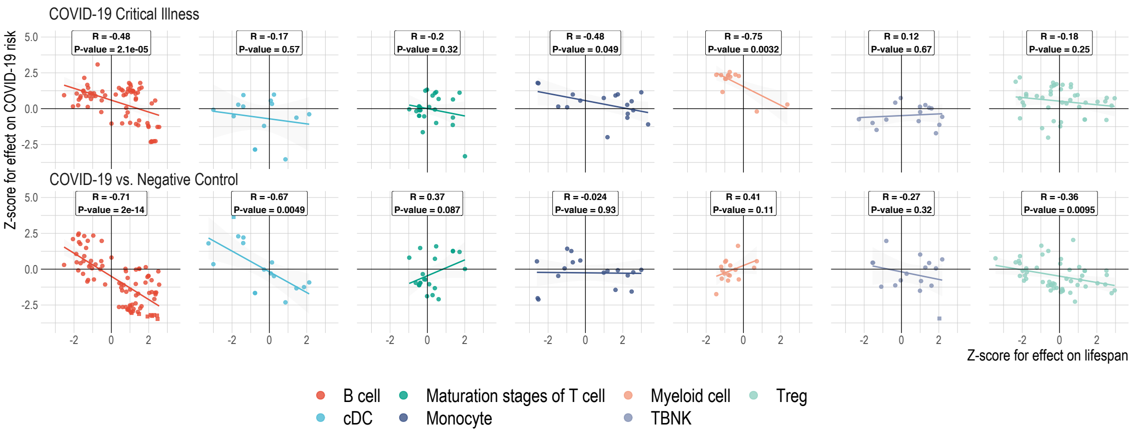

Figure S7. Negative correlation between the effect of immune cell traits on lifespan and COVID-19 risk in different immune cell type panels. Mendelian Randomization results showing the effect of immune cell surface marker levels on lifespan (x-axis); the y-axis shows the Z-score for the risk of COVID-19 infection and COVID-19 critical illness. Each panel represent a cell type. Traits with FDR < 0.05 for at least one outcome are shown in squares.

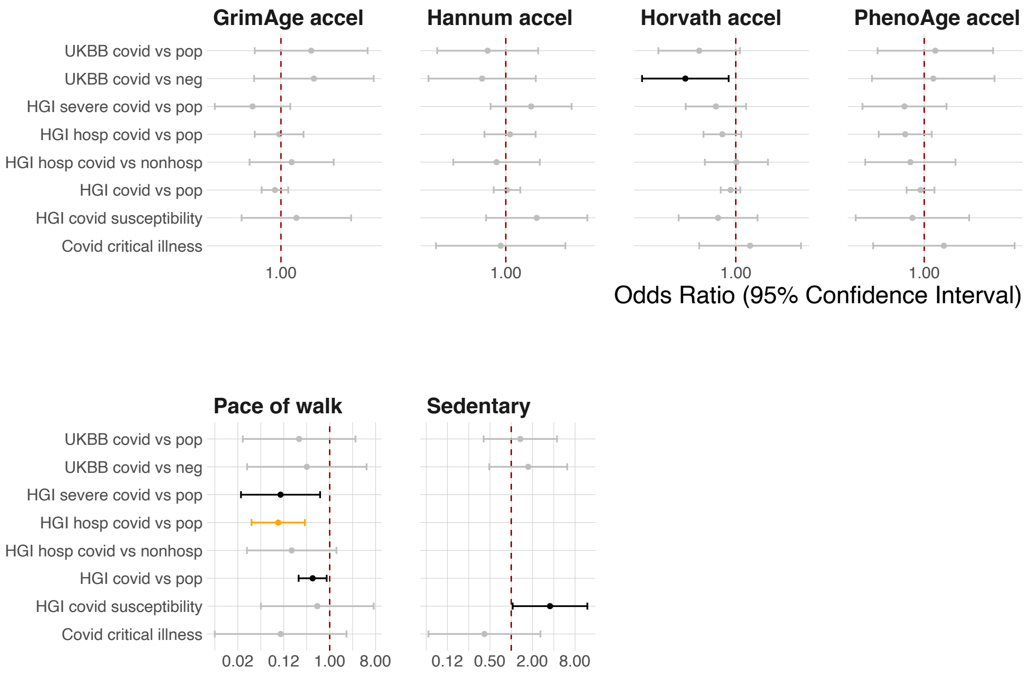

Figure S8. Mendelian randomization analysis investigating the association of genetically proxied epigenetic age acceleration and physical activity with the risk of COVID-19. Forest plots showing Mendelian randomization estimates for the causal effect of epigenetic age acceleration and physical activity on the risk of COVID-19. Error bars show the 95% confidential interval. Significant effects with FDR < 0.05 are in orange. Nominally significant effects ($P < 0.05$) are in black.

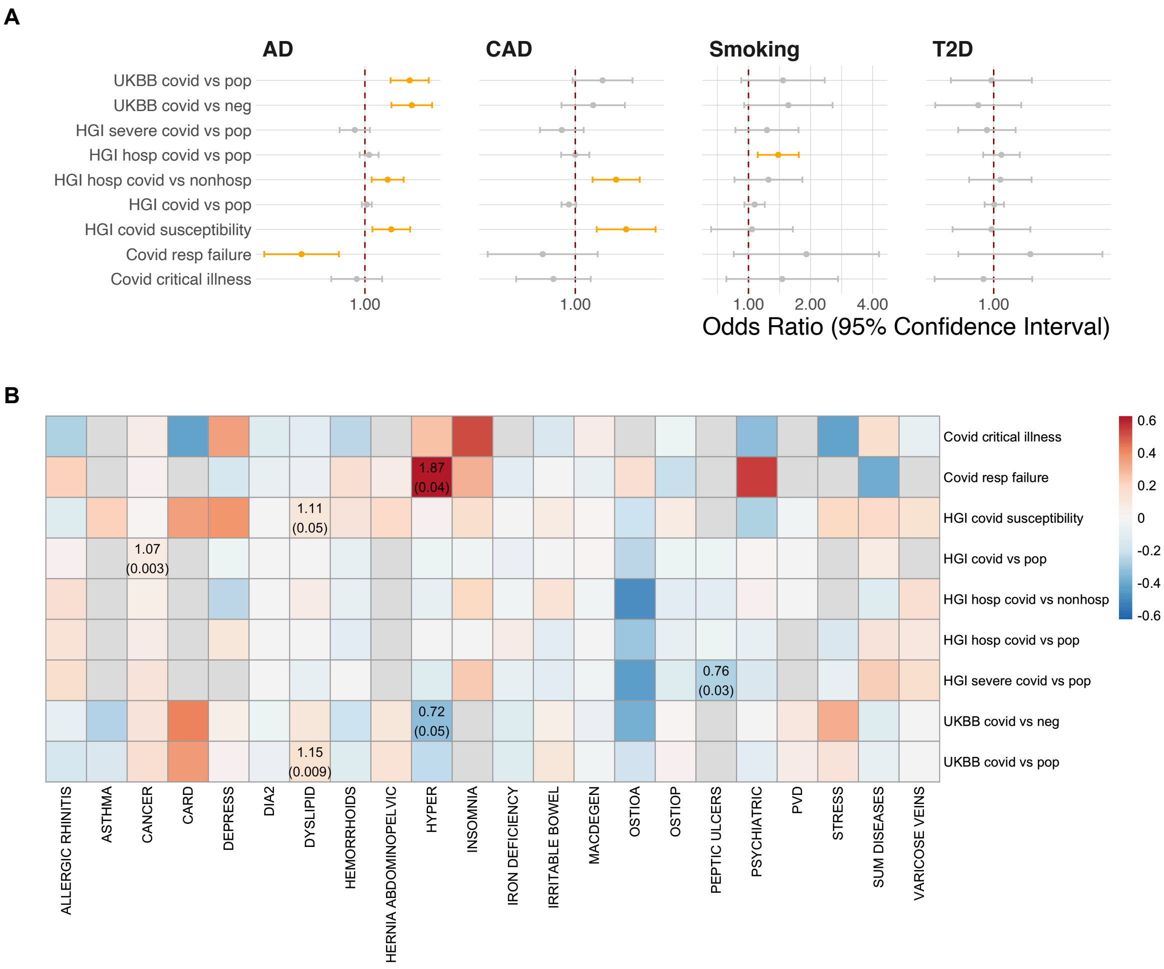

Figure S9. Putative causal associations between diseases and the risk of COVID-19. A. Forest plots showing Mendelian randomization estimates for the causal effect of lifespan-related risk factors on the risk of COVID-19. Error bars show the 95% confidential interval. Significant effects with FDR < 0.05 are in orange. Nominally significant effects (P < 0.05) are in black. **B.** Shown are the results of GSMR analyses with disease data from a community-based study (GERA). Colors represent the effect sizes (as measured by log odds ratios, log ORs) of diseases on COVID-19, red for risk effects, blue for protective effects, and gray means the effect cannot be estimated due to limited available SNPs. Nominally significant effects ($P < 0.05$) are labeled with OR (P-value).

Table S1. Exposure and outcome trait genetic summary data sources

Trait	Abbreviation	Ancestry	N ($N_{cases}/N_{controls}$)	Publication (source)	URLs
**Healthspan**	Healthspan	European	300,447	Zenin *et al.* 2019 *Communications biology*	https://zenodo.org/record/1302861/files/healthspan_summary.csv.gz?download=1
**Lifespan**	Lifespan	European	1,012,240	Timmers *et al.* 2019 *Elife*	https://datashare.is.ed.ac.uk/bitstream/handle/10283/3209/lifegen_phase2_bothpl_alldr_2017_09_18.tsv.gz?sequence=1&isAllowed=y
**Longevity (age >90th survival percentile)**	Longevity	European	11,262/25,483	Deelen *et al.* 2019 *Nature Communications*	https://www.longevitygenomics.org/downloads
**Meta-analysis of Healthspan, Lifespan and Longevity**	Healthy aging	European	-	Timmers *et al.* 2020 *Nature Communications*	https://datashare.is.ed.ac.uk/bitstream/handle/10283/3599/timmers2020_healthspan_lifespan_longevity.tsv.gz?sequence=2&isAllowed=y
**Hannum age**	Hannum_accel	European	34,449	McCartney *et al.* 2020 *BioRxiv*	https://datashare.is.ed.ac.uk/handle/10283/3645
**Horvath age**	Horvath_accel				
**PhenoAge**	PhenoAge_accel				
**GrimAge**	GrimAge_accel				
**SARS-COV-2 infection Positive vs. Population**	UKBB_covid_vs_pop	European	1,503 / 457,747	Genome-Wide Repository of Associations Between SNPs and Phenotypes	https://grasp.nhlbi.nih.gov/downloads/COVID19GWAS/08042020/UKBB_covid19_EUR_080420.txt.gz
**SARS-COV-2 infection Positive vs. Negative (tested)**	UKBB_covid_vs_neg	European	1,503 / 10,632	Genome-Wide Repository of Associations Between SNPs and Phenotypes	https://grasp.nhlbi.nih.gov/downloads/COVID19GWAS/08042020/UKBB_covid19_EURtested_080420.txt.gz
**Critical illness of COVID-19**	Covid critical illness	Mixed	2,244	Pairo-Castineira, E. *et al.* 2020 *Nature*	
**Hospitalized covid vs. not hospitalized covid**	HGI_hosp_covid_vs_nonhosp	Mixed	5,773/15,497	The COVID-19 host genetics initiative	https://storage.googleapis.com/covid19-hg-public/20201215/results/20210107/COVID19_HGI_B1_ALL_leave_23andme_20210107.b37.txt.gz
**Hospitalized covid vs. population**	HGI_hosp_covid_vs_pop	Mixed	12,888/1,295,966	The COVID-19 host genetics initiative	https://storage.googleapis.com/covid19-hg-public/20201215/results/20210107/COVID19_HGI_B2_ALL_leave_23andme_20210107.b37.txt.gz
**Susceptibility (affected vs. population)**	HGI_covid_susceptibility	EUR, FIN, SAS, CEU, AFR	1,678/674,635	The COVID-19 host genetics initiative	https://storage.googleapis.com/covid19-hg-public/20200508/results/COVID19_HGI_ANA5_20200513.txt.gz
**Very severe respiratory confirmed covid vs. population**	HGI_severe_covid_vs_pop	Mixed	5,582/709,010	The COVID-19 host genetics initiative	https://storage.googleapis.com/covid19-hg-public/20201215/results/20210107/COVID19_HGI_A2_ALL_leave_23andme_20210107.b37.txt.gz
**Covid vs. population**	HGI_covid_vs_pop	Mixed	36,590/1,668,938	The COVID-19 host genetics initiative	https://storage.googleapis.com/covid19-hg-public/20200619/results/build_37/COVID19_HGI_ANA_C2_V2_20200701.b37.txt.gz

Table S2. Detecting pleiotropic effect of lifespan-related traits based on intercept term in MR Egger regression

Exposure	Outcome	Egger intercept	SE	P
Healthy aging	HGI covid susceptibility	0.02	0.02	0.36
Healthy aging	HGI hosp covid vs nonhosp	0.00	0.02	0.98
Healthy aging	HGI hosp covid vs pop	-0.01	0.02	0.71
Healthy aging	UKBB covid vs neg	0.02	0.03	0.50
Healthy aging	UKBB covid vs pop	0.03	0.03	0.24
Lifespan	HGI covid susceptibility	0.02	0.04	0.66
Lifespan	HGI hosp covid vs nonhosp	0.01	0.04	0.88
Lifespan	HGI hosp covid vs pop	-0.01	0.02	0.71
Lifespan	UKBB covid vs neg	0.05	0.04	0.23
Lifespan	UKBB covid vs pop	0.07	0.03	0.04
Longevity	HGI covid susceptibility	-0.08	0.36	0.85
Longevity	HGI hosp covid vs nonhosp	-0.17	0.15	0.45
Longevity	HGI hosp covid vs pop	-0.11	0.08	0.39
Longevity	UKBB covid vs neg	-0.30	0.43	0.56
Longevity	UKBB covid vs pop	-0.25	0.44	0.62

Table S3. Detecting pleiotropic effect of Notch eQTLs based on intercept term in MR Egger regression

Exposure	Outcome	Egger intercept	SE	P
NOTCH2	UKBB covid vs neg	-0.05	0.04	0.18
NOTCH2	UKBB covid vs pop	-0.06	0.04	0.14

Table S4. Common diseases in GERA cohort

Common Disease	***Abbreviation***	***N_cases***	***N_controls***
Asthma	ASTHMA	10,080	51,767
Allergic Rhinitis	ALLERGIC_RHINITIS	15,166	46,681
Cardiovascular Disease	CARD	16,399	45,448
Cancer	CANCER	18,677	43,170
Major Depressive Disorder	DEPRESS	7,892	53,955
Dermatophytosis	/	8,428	53,419
T2D	DIA2	7,624	54,223
Dyslipidemia	DYSLIPID	33,024	28,823
Hypertensive Disease	HYPER	31,000	30,847
Hemorrhoids	HEMORRHOIDS	9,898	51,949
Hernia Abdominopelvic Cavity	HERNIA_ABDOMINOPELVIC	6,864	54,983
Insomnia	INSOMNIA	4,346	57,501
Iron Deficiency Anemias	IRON_DEFICIENCY	2,699	59,148
Irritable Bowel Syndrome	IRRITABLE_BOWEL	3,359	58,488
Macular Degeneration	MACDEGEN	4,026	57,821
Osteoarthritis	OSTIOA	22,022	39,825
Osteoporosis	OSTIOP	5,898	55,949
Peripheral Vascular Disease	PVD	4,708	57,139
Peptic Ulcer	PEPTIC_ULCERS	1,004	60,843
Psychiatric Disorder	PSYCHIATRIC	9,394	52,453
Acute Reaction to Stress	STRESS	4,695	57,152
Varicose Veins	VARICOSE_VEINS	2,711	59,136
