# Supplementary material for: Genetic and phenotypic analysis of the causal relationship between aging and COVID-19
Source: Commun Med (Lond). 2021 Oct 5;1:35. doi: 10.1038/s43856-021-00033-z (PMC9053191; doi:10.1038/s43856-021-00033-z)
Supplement: Supplementary file 10 — Reporting Summary [file 43856_2021_33_MOESM10_ESM.pdf]

nature research

Corresponding author(s): Xia Shen, Vadim N. Gladyshev

Last updated by author(s): Aug 24, 2021

Reporting Summary

Nature Research wishes to improve the reproducibility of the work that we publish. This form provides structure for consistency and transparency in reporting. For further information on Nature Research policies, see our Editorial Policies and the Editorial Policy Checklist.

Statistics

For all statistical analyses, confirm that the following items are present in the figure legend, table legend, main text, or Methods section.

n/a	Confirmed	
☐	☒	The exact sample size (*n*) for each experimental group/condition, given as a discrete number and unit of measurement
☐	☒	A statement on whether measurements were taken from distinct samples or whether the same sample was measured repeatedly
☐	☒	The statistical test(s) used AND whether they are one- or two-sided<br>*Only common tests should be described solely by name; describe more complex techniques in the Methods section.*
☐	☒	A description of all covariates tested
☐	☒	A description of any assumptions or corrections, such as tests of normality and adjustment for multiple comparisons
☐	☒	A full description of the statistical parameters including central tendency (e.g. means) or other basic estimates (e.g. regression coefficient) AND variation (e.g. standard deviation) or associated estimates of uncertainty (e.g. confidence intervals)
☐	☒	For null hypothesis testing, the test statistic (e.g. *F*, *t*, *r*) with confidence intervals, effect sizes, degrees of freedom and *P* value noted<br>*Give P values as exact values whenever suitable.*
☒	☐	For Bayesian analysis, information on the choice of priors and Markov chain Monte Carlo settings
☒	☐	For hierarchical and complex designs, identification of the appropriate level for tests and full reporting of outcomes
☐	☒	Estimates of effect sizes (e.g. Cohen's *d*, Pearson's *r*), indicating how they were calculated

Our web collection on statistics for biologists contains articles on many of the points above.

Software and code

Policy information about availability of computer code

Data collection	The genome-wide association summary-level data was obtained via publicly available links online. The individual phenotypic data was obtained by application to UK Biobank.
Data analysis	The GSMR analysis was performed using GCTA 1.93.1beta: https://cnsgenomics.com/software/gcta/<br>All results was analyzed using R 4.0.2: https://www.r-project.org/.<br>The bivariate genomic scan was performed using R package "MultiABEL": https://github.com/xiashen/MultiABEL<br>The pathway enrichment analysis for GWAS summary statistics was performed on FUMA platform: https://fuma.ctglab.nl/<br>The genetic correlation analysis was performed using: LDSC v1.0.1: https://github.com/bulik/ldsc and HDL v1.3.8: https://github.com/zhenin/HDL

For manuscripts utilizing custom algorithms or software that are central to the research but not yet described in published literature, software must be made available to editors and reviewers. We strongly encourage code deposition in a community repository (e.g. GitHub). See the Nature Research guidelines for submitting code & software for further information.

Data

Policy information about availability of data

All manuscripts must include a data availability statement. This statement should provide the following information, where applicable:

- Accession codes, unique identifiers, or web links for publicly available datasets
- A list of figures that have associated raw data
- A description of any restrictions on data availability

GWAS summary statistics used in this study are publicly available (for URLs, see Table S1). The individual-level phenotype data are available in the UK Biobank (http://www.ukbiobank.ac.uk/) upon application and with permission of UKBB's Research Eth-ics Committee. The source data for the main figures can be accessed

as Supplementary Data 2-6. The bivariate GWAS summary statistics of aging-related COVID-19 generated in this study are available on Figshare (https://figshare.com/articles/dataset/combined_ukbbCOVID_meta_txt/16416822).

Field-specific reporting

Please select the one below that is the best fit for your research. If you are not sure, read the appropriate sections before making your selection.

☒ Life sciences ☐ Behavioural & social sciences ☐ Ecological, evolutionary & environmental sciences

For a reference copy of the document with all sections, see nature.com/documents/nr-reporting-summary-flat.pdf

Life sciences study design

All studies must disclose on these points even when the disclosure is negative.

Sample size	The work described in this manuscript uses summary statistics from previously conducted genome-wide association studies and the phenotype data from the UK Biobank cohort. Therefore, we do not control the sample size for these studies. Sample sizes for all summary statistics included are given in Supplementary Tables S1 and S3. Sample sizes for the UK Biobank traits are shown in the Figure 2 and S2.
Data exclusions	Duplicated markers were excluded from the summary statistics prior to the analysis. We didn't exclude any sample from the UK Biobank dataset.
Replication	The results are reproducible by re-running the analysis pipeline. No additional replication studies was applicable.
Randomization	The work used publicly available summary statistics and UK Biobank observational data, therefore no randomization was applied.
Blinding	The investigators had no involvement in data collection. No group allocation was applicable.

Reporting for specific materials, systems and methods

We require information from authors about some types of materials, experimental systems and methods used in many studies. Here, indicate whether each material, system or method listed is relevant to your study. If you are not sure if a list item applies to your research, read the appropriate section before selecting a response.

Materials & experimental systems

n/a	Involved in the study	
☒	☐	Antibodies
☒	☐	Eukaryotic cell lines
☒	☐	Palaeontology and archaeology
☒	☐	Animals and other organisms
☐	☒	Human research participants
☒	☐	Clinical data
☒	☐	Dual use research of concern

Methods

n/a	Involved in the study	
☒	☐	ChIP-seq
☒	☐	Flow cytometry
☒	☐	MRI-based neuroimaging

Human research participants

Policy information about studies involving human research participants

Population characteristics	The data used in this work are summary statistics from 46 genome wide association studies and phenotypic information from the UK Biobank cohort. These studies were all conducted in adults. The detailed ancestry information are provided in Supplementary Table S1.
Recruitment	We did not recruit the study participants.
Ethics oversight	Besides the publicly available summary-level data, ethical approval was provided by the UK Biobank cohort.

Note that full information on the approval of the study protocol must also be provided in the manuscript.
