# Supplementary material for: Genetic and phenotypic analysis of the causal relationship between aging and COVID-19
Source: Commun Med (Lond). 2021 Oct 5;1:35. doi: 10.1038/s43856-021-00033-z (PMC9053191; doi:10.1038/s43856-021-00033-z)
Supplement: Supplementary file 11 — Description of Additional Supplementary Files [file 43856_2021_33_MOESM11_ESM.pdf]

Description of Additional Supplementary Files

File Name: Supplementary Data 1

Description: MR instruments for exposure traits

File Name: Supplementary Data 2

Description: MR result summary

File Name: Supplementary Data 3

Description: Biological age acceleration and future COVID-19 risk

File Name: Supplementary Data 4

Description: Gene set enrichment result for bivariate genomic scan

File Name: Supplementary Data 5

Description: MR result of immune cell traits on lifespan and COVID-19

File Name: Supplementary Data 6

Description: COVIDIP CD19 level on B cells in COVID-19 patients

File Name: Supplementary Data 7

Description: Genetic correction result

File Name: Supplementary Data 8

Description: Lifespan-COVID-19 bivariate genomic scan top SNPs
